# Implementation of Modified Effective Butterfly Optimizer in Solving Multi-Objective Pareto Optimal Power Flow Problem with Renewable Uncertainties

**DOI:** 10.3390/biomimetics11060418

**Published:** 2026-06-13

**Authors:** Hakan Işıker, Ali Akdağlı, Volkan Yamaçlı, Zeki Yetgin, İbrahim Çağrı Barutçu, Kadir Abacı, Furkan Gözükara

**Affiliations:** 1 Electrical & Electronics Engineering Department, Faculty of Engineering, Mersin University, Mersin 33100, Turkey; hakan.isiker@mersin.edu.tr (H.I.); kabaci@mersin.edu.tr (K.A.); 2 Computer Engineering Department, Faculty of Engineering, Mersin University, Mersin 33100, Turkey; vyamacli@mersin.edu.tr (V.Y.); zyetgin@mersin.edu.tr (Z.Y.); furkangozukara@mersin.edu.tr (F.G.); 3 Electricity and Energy Department, Hakkari University, Hakkari 30000, Turkey; ibrahimcagribarutcu@hakkari.edu.tr

**Keywords:** modified effective butterfly optimizer, bio-inspired optimization, Pareto optimization, optimal power flow, renewable uncertainty

## Abstract

The power flow problem is one of the most challenging tasks in power systems, affecting both generation cost and energy quality. Optimal power flow (OPF) further complicates this task by requiring the optimal adjustment of system variables and parameters. This paper adapts the Modified Effective Butterfly Optimizer (MEBO) to solve multi-objective optimal power flow (MOOPF) problems with the contribution of optimized weighting using multiple Pareto archives. MEBO is an advanced optimization algorithm that utilizes population reduction and parameter learning to guide subsequent searches for unconstrained problems. The proposed technique has been tested on IEEE 30 and 57 bus test systems, and the results have been compared with existing methods reported in the literature. In the paper, four single-objective functions, namely generator cost, active power loss, fuel emission, and voltage deviation, are used to construct four multi-objective (MO) problems: cost–loss, cost–voltage, cost-emission, and emission–loss. For the cost-emission case, the proposed MEBO achieved compromised solutions of 791.1951 $/h fuel cost with 0.10873 ton/h emission and 801.8172 $/h fuel cost with 0.10044 ton/h emission under different Pareto-based optimization metrics. In the emission–loss case, the algorithm obtained 0.20539 ton/h emission with 3.1403 MW/h power loss, demonstrating the effectiveness of the proposed approach in balancing conflicting objectives. The Pareto curves of MEBO in achieving MO problems are presented, along with the suggested compromised solutions acquired from the literature. In the literature, this is the first application of MEBO for solving MOOPF problems. The results demonstrate that MEBO performs better than most other alternatives; this shows potential for further improvements with respect to the MOOPF problem.

## 1. Introduction

An electric power system is a comprehensive arrangement of components designed to supply, transfer, and consume electricity. It consists of three main components: the distribution system, which serves homes and businesses; the transmission system, which transports electricity from generating centers to load centers; and the generators, which produce power. Recently, optimizing system parameters for economic and environmental reasons has become increasingly important. As a result, the use of computer simulations for large-scale power systems—serving entire countries or cities—has become essential, leading to the development of the concept of “optimal power flow” (OPF).

OPF poses a critical and intricate optimization challenge within electric power systems. It is focused on determining the best configurations for controllable parameters within a power network to achieve specific objectives while upholding operational constraints. Since its inception in 1962 [1], OPF has remained a widely explored topic in power system networks. While the traditional aim of OPF solutions is to minimize fuel costs, utility providers also have commercial considerations, and the reduction of transmission losses is vital for maintaining high power quality. Furthermore, in response to increasing environmental concerns, the optimization of emission levels as an objective function rather than a mere constraint is necessary [2]. Consequently, researchers are increasingly interested in the multi-objective optimal power flow (MO-OPF) problem, which addresses the simultaneous optimization of multiple objective functions.

Multi-objective (MO) optimization in power systems introduces complexity into decision-making process, demanding the simultaneous consideration of multiple conflicting goals. It is an essential tool for power system operators and planners, enabling them to balance diverse and often competing objectives such as cost minimization, emission reduction, and system reliability. To tackle this versatile optimization problem, researchers have proposed various techniques.

In the literature, there are two common approaches to developing the best MO solution to minimize multiple objective functions: classical methods and studies on multi-objective evolutionary algorithms (MOEAs) [3]. Traditional optimization procedures yield a single solution point, whereas MO methods provide a set of optimal solutions, known as the Pareto set. Traditional methods convert an MO problem into a single-objective problem by assigning a suitable weighting factor to each objective. The aim is to establish a relationship between the range of each objective function and its associated weight. For instance, if the range of an objective function is large, it should be assigned a small weight [4]. By adhering to this relationship, decision-makers can ascertain appropriate weights. The optimal weight is determined based on the maximum range of each objective function. When the optimal weight is applied to the MO function comprising the cost of generation and transmission losses, the target point can be achieved in a single step, thereby reducing the computational effort required [5].

Many studies in the literature use weight coefficients to relate objective functions to each other. In [6], the modified weighted teaching-learning based optimization (WTLBO) algorithm was utilized to transform MO functions into a single-objective function using the weighted sum method. In [7], the Artificial Bee Colony (ABC) algorithm is employed to solve the OPF problem, considering both equality and inequality constraints in an electric power system. Ref. [8] not only reduces fuel costs for generators but also improves the voltage profile and minimizes active power loss. In [9], the chaotic equilibrium optimization (CEO) algorithm is applied to the single and MO-OPF problems, incorporating weights for the objective functions in the solution. Another approach to address the MO-OPF problem with the weight coefficient is proposed in [10]. The study introduces the Improved Archimedes Optimization Algorithm (IAOA) and applies it to single and MO targets, comparing it with other techniques in the literature. In addition to these studies, improved moth flame optimization (IMFO) [11] uses the weight coefficient, and the differential search algorithm (DSA) [12] and improved colliding bodies optimization algorithm (ICBO) [13] are used to solve the MO-OPF problem. The hybrid algorithms presented in [14,15] have effectively addressed single and MO-OPF problems in the 30- and 118-bus IEEE systems by utilizing a weighting method. Notably, the hybrid algorithm (AO–AOA), which combines the Aquila optimizer (AO) and the arithmetic optimization algorithm (AOA), has demonstrated considerable success in solving various objective functions, as outlined in [14]. Ref. [15] introduced the hybrid (HRSCA) algorithm to the literature for tackling the OPF problem; this algorithm enhances solution quality by merging the exploratory capabilities of the sine-cosine algorithm (SCA) with the robust exploitation ability of the Rao-2 algorithm. There is also growing interest in addressing the MO-OPF problem by using the weighting method in power systems that incorporate renewable energy sources. In [16], the hybrid [HBOA] algorithm, which integrates the strengths of the hippopotamus algorithm and the brown bear solver through QOBL, was introduced to the field.

These approaches are straightforward to implement and can be employed for single-objective algorithms without modification. However, these algorithms can only identify an optimal solution in a single simulation run of the algorithm. Consequently, these methods cannot identify the compromises between multiple objectives. To overcome these disadvantages, early Multi-Objective Optimization Algorithms (MOEAs) developed various new and effective methods for addressing the Multi-Objective Optimization Front (MO-OPF) problem, aiming to accurately depict the Pareto optimal front and offer a wider range of solutions for decision-makers. In [17], researchers utilized the shuffle frog leaping algorithm (SFLA) to address the MO-OPF problem, with a focus on emissions. To overcome the challenge of tuning control parameters, the authors of [18] proposed an enhanced self-adaptive DE and a mix crossover operator (ESDE-MC) capable of handling both single- and MO-OPF problems. Another approach, described in [2], introduced the MO evolutionary algorithm-based decomposition (MOEA/D), using a modified Tchebycheff decomposition method to obtain uniformly distributed Pareto optimal solutions on each objective space. Additionally, a technique called the quasi-oppositional teaching learning-based optimization (QOTLBO) algorithm was introduced in [19], which involves integrating the quasi-oppositional learning algorithm into the teaching learning-based optimization (TLBO) algorithm to enhance convergence speed and solution quality. Furthermore, the authors of [20] recommended a modified TLBO (MTLBO) to address the MO-OPF problem with emission and cost objectives. In addition to these advancements, various Pareto concept-based metaheuristic techniques have been implemented, such as the MOEAs based on decomposition superiority of feasible solutions (MOEA/D-SF) [21] and the interior search algorithm (ISA) [22].

Recent studies further confirm the continuing methodological evolution of MO-OPF. Khunkitti et al. proposed an SMA-based MO-OPF method in which Pareto dominance stores non-dominated solutions and a crowding mechanism manages the Pareto repository; the method was tested on the IEEE 30-, 57-, and 118-bus systems [23]. Al-Kaabi et al. developed a Hunger Games Search-based MO-OPF framework (MOHGS) that optimizes fuel cost, active power loss, emission, voltage deviation, and voltage stability index by combining Pareto optimization, fuzzy-membership-based best compromise selection, and crowding-distance ranking [24]. Likewise, Mallala et al. reported a non-dominated sorting hybrid fruit-fly-based ABC method for MO-OPF, considering generation cost, transmission power loss, and severity value, and using a fuzzy decision mechanism to select a compromise solution from the Pareto set on the IEEE 30-bus system [25]. Related contributions also include: the sustainability study by Diab et al., which proposed a multi-objective OPF control technique based on MVO, GOA, and HHO, validated on the IEEE 30-bus system and compared with PSO [26]; the processes paper by Wu et al., which proposed an improved MOEA/D (IMOEA/D) with three enhancement strategies and tested it on the IEEE 30- and 57-bus systems [27]; the recent energies study by Alsokhiry, which applied HHO to a six-objective OPF formulation on the IEEE 30-bus system and benchmarked it against MOEA/D-DRA and NSGA-III [28]; and the applied system innovation paper by ElMessmary et al., which introduced Egyptian Stray Dog Optimization (ESDO) for both single- and multi-objective OPF on the IEEE 30-bus system [29]. In addition, Hakmi et al. applied Honey Badger Optimization to OPF on the IEEE 30-bus system and explicitly analyzed the Pareto trade-off between fuel cost and voltage deviation [30]. Beyond deterministic formulations, recent studies have emphasized the explicit treatment of renewable uncertainty in OPF. The authors of this manuscript [31,32] have studied OPF in systems incorporating renewable sources such as wind and photovoltaic generation. In one study, well-known IEEE power systems were optimized with wind power under a single-objective formulation [31], while in another, IEEE power systems, including both wind and photovoltaic resources, were optimized with a focus on real-world and practical concerns [32]. Shaheen et al. formulated a probabilistic OPF using stochastic models for solar PV and wind generation while also accounting for load-demand variations [33]. Another study [34] introduced the multi-objective (MOSAWGA) algorithm to the literature by extending the classical wild geese algorithm (WGA) to enhance its search capabilities in a power system incorporating photovoltaic and wind units. Li et al. [35] approached the multi-objective optimal power flow (MO-OPF) problem by incorporating random wind, photovoltaic, and tidal sources through a multi-objective pathfinder algorithm, noting that the integration of renewables in OPF remains in its early stages. Al-Kaabi et al. [36] developed Pareto-based multi-objective variants of the grey wolf optimizer (GWO) and Harris hawks optimization (HHO) for the IEEE 30-bus and IEEE 57-bus test cases.

Relatedly, Nazari and Abdi applied imperialist competitive Harris hawks optimization to address the combined heat and power economic dispatch problem across different-scale power systems [37]. In contrast, ALBaaj and Kaplan [38] explored uncertain scenarios of renewable distributed generation (DG) using an enhanced COVID-19 optimization algorithm. Furthermore, Katkar and Jadhav [39] pointed out existing gaps, including inadequate probabilistic modeling of renewables, challenges in constraint handling, the need to preserve Pareto-front diversity, and biases in post-Pareto decision analysis within renewable-integrated MOOPF. Alghamdi investigated OPF in hybrid wind/solar/thermal systems under objectives including fuel cost and emissions, and additionally examined voltage-profile improvement, power-loss reduction, valve-point effects, and carbon-tax cases [40]. Abid et al. further incorporated solar PV, wind, and hydropower sources together with FACTS devices into a multi-objective OPF framework; in the renewable-source modeling, wind, solar, and hydropower uncertainties were represented by Weibull, lognormal, and Gumbel distributions, respectively [41]. In addition, Pandey et al. proposed a Golden Jackal optimization (GJO) approach for the optimal management of a virtual power plant to reduce reliance on fossil fuels using single and multi-objective functions [42]; building on the same optimizer, Jiang et al. introduced the SCMGJO variant, which integrates tent-map reverse learning, sine-cosine search, and Cauchy mutation, and validated it on 23 benchmark functions and three engineering design problems [43].

Recent studies further reinforce the renewable-integrated perspective adopted in this analysis. Khamees et al. modeled wind speed and solar irradiance using a three-component mixture distribution and applied the Mayfly algorithm to address both single- and multi-objective optimal power flow (OPF) cases on the IEEE 30-bus system [44]. In a subsequent study, Zhu et al. [45] advanced the Mayfly algorithm by enhancing the processing of non-dominant ranking and superiority constraints for feasible solutions in the integrated wind and solar energy contexts of the IEEE 30 and 57 bus systems. Alanazi et al. incorporated photovoltaic and wind-turbine units into the OPF formulation, treating estimated PV/WT output powers as dependent variables and PV/WT bus-voltage magnitudes as control variables; real-time wind-speed and irradiance data were used to estimate and predict renewable generation [46]. More recently, Lu et al. introduced the Calibrated Safety Constraints Optimal Power Flow (CSCOPF) model, which employs an enhanced acceleration coefficient-based bee swarm algorithm. This model incorporates both preventive safety planning and power event safety analysis for wind energy integrated systems, alongside an equivalent current injection model [47]. Related studies reinforce this probabilistic viewpoint. Riaz et al. developed a hybrid PSO–GWO-based cost-oriented OPF strategy for systems integrated with stochastic solar photovoltaic and wind generation [48], and Shaheen et al. later combined machine learning with transient search optimization for probabilistic OPF under renewable and time-varying load uncertainty [49]. Complementary recent studies have also diversified the solution landscape. Yamaguti et al. formulated a stochastic scenario-based economic/environmental multi-objective convex OPF model using SOCP and an ε-constrained algorithm to handle generation cost, active power loss, and emissions under RES and load uncertainties [50].

The MO-OPF problem has been extensively studied in recent years using both classical formulations and MO Pareto-based solutions. Various metaheuristic algorithms have been successfully applied for both approaches. In Pareto-based methods, the solution is not a single point but a set of non-dominated alternatives covering the trade-off surface. Consequently, the decision-maker must select the most suitable solution from a wide range of candidates, which creates an additional challenge in identifying the final compromised solution because different runs of the application typically result in different compromise solutions. In addition, although many recent MOEAs have demonstrated strong optimization capability, several studies in the literature still report shortcomings related to Pareto-front diversity preservation, constraint-handling robustness, computational complexity, and post-Pareto decision analysis, especially in renewable-integrated MO-OPF problems. Therefore, there remains a need for robust optimization frameworks supporting effective compromise solution selection. Pareto-based algorithms such as NSGA-type or non-dominated-sorting variants often require additional archive, crowding-distance, or dominance mechanisms, which may increase computational cost and parameter sensitivity. In the Pareto-based approaches, renewable uncertainty is frequently represented through fixed probability distributions or limited scenarios, while the correlation among wind, solar, load, and reserve/penalty costs is not always examined. Many studies focus on reporting algorithm-by-algorithm numerical superiority, whereas fewer studies discuss how the final best compromise solution should be selected from the Pareto set. These limitations show the need for a lightweight yet adaptive algorithmic framework with explicit decision-maker metrics for selecting compromise solutions. Motivated by these research gaps, this study investigates the applicability of the Modified Effective Butterfly Optimizer (MEBO) which forms part of the EBOwithCMAR framework [51], for solving constrained MO-OPF problems under different objective combinations. This line of work is relevant here because MO-OPF with renewable uncertainty remains a high-dimensional constrained search problem in which exploration–exploitation balance, diversity preservation, and constraint handling strongly influence the quality of the obtained Pareto set. To overcome these problems, this study proposes a novel weighting approach with Modified EBO where the exploit areas are first discovered using multiple shallow (quick) searches and then optimum area is deeply searched after finding the optimum weight. Each search is associated with a weight and an independent Pareto archive to keep the potential front points. Finally, the proposed method combines the Pareto archives and utilizes distance measurement techniques such as Euclidean, Mahalanobis, and City Block to identify the optimal compromise solution on the acquired Pareto front. The proposed method addresses the classical MO problem of objective functions, including fuel cost, fuel emissions, voltage deviation, and active power loss.

The main contributions of this paper are as follows:

- adaptation of MEBO to solve a complex MO-OPF system including renewable energy using optimized weighting with multiple Pareto archives;
- a novel selection method for a compromised solution using multiple evaluation distances;
- the performance comparison of the proposed approach with the recent state-of-the-art studies in the literature.

The remainder of this study is organized as follows: Section 2 formulates the MO-OPF problem; Section 3 describes the proposed WSMOPF program technique with weight strategy and explains the proposed MEBO and its implementation; Section 4 presents the simulation results; and Section 5 concludes the study.

## 2. Formulation of the Multi-Objective OPF Problem

In this MO-OPF model, the task is expressed as a constrained vector-optimization problem. The optimizer adjusts the available control variables so that several performance indices are improved at the same time, while the network power-balance equations and all operating bounds remain satisfied.

The vector form of the multi-objective search can be written as

$$\Psi \left(s,c\right)=\left[f^{1}\left(x,u\right),\;\;f^{2}\left(x,u\right),\ldots ,f^{M}\left(x,u\right)\right]$$

subject to the equality and inequality restrictions

$$g\left(x,u\right)=0,\;\;h\left(x,u\right)\leq 0$$

where $f^{r}\left(x,u\right)$ is the *r*th objective component, M is the number of objectives, $g\left(\cdot \right)$ denotes the power-balance equations, and $h\left(\cdot \right)$ collects the operational limits. The state vector used by the load-flow solution is defined as

(1) $$x^{T}=\left[P_{ref},\;V_{PQ},\;Q_{G},\;S_{br}\right]$$

Here, $P_{ref}$ is the active generation assigned to the reference bus, $V_{PQ}$ contains the voltages of load buses, $Q_{G}$ gives generator reactive outputs, and $S_{br}$ represents branch loading quantities. The controllable decision vector is arranged as

(2) $$u^{T}=\left[P_{G},\;V_{G},\;Q_{sh},\;\tau \right]$$

where $P_{G}$ and $V_{G}$ are the active-power dispatches and voltage set-points of generator buses, respectively. The vector $Q_{sh}$ denotes shunt compensation settings and τ denotes on-load tap-changer ratios. Thus, the feasible OPF search space is determined by the AC power-flow residuals together with equipment ratings, bus-voltage limits, and line-loading restrictions.

### 2.1. Load-Flow Equations and Constraints

For each bus k, the active- and reactive-power mismatch equations are expressed in residual form as

(3) $$P_{k}=P_{G,k}-P_{D,k}-\sum_{r=1}^{N_{b}}\left|V_{k}\right|\left|V_{r}\right|\left|Y_{kr}\right|cos\left(\theta_{kr}-\delta_{k}+\delta_{r}\right)=0$$

(4) $$\Delta Q_{k}=Q_{G,k}-Q_{D,k}-\sum_{r=1}^{N_{b}}\left|V_{k}\right|\left|V_{r}\right|\left|Y_{kr}\right|sin\left(\theta_{kr}-\delta_{k}+\delta_{r}\right)=0$$

In these expressions, $P_{G,k}$ and $Q_{G,k}$ denote the generated active and reactive powers at bus k, while $P_{D,k}$ and $Q_{D,k}$ denote the corresponding load demands. The magnitude and angle of the admittance element between buses k and r are represented by $\left|Y_{kr}\right|$, $\theta_{kr}$, $\delta_{k}$ is the voltage phase angle, and $N_{b}$ is the total number of buses. The remaining feasibility conditions are given below.

#### 2.1.1. Generator Constraints

Each generator is operated inside its admissible voltage, active-power, and reactive-power intervals:

(5a) $${\underline{V}}_{G,i}\leq V_{G,i}\leq {\bar{V}}_{G,i},\;\;i\in G$$

(5b) $${\underline{P}}_{G,i}\leq P_{G,i}\leq {\bar{P}}_{G,i},\;\;i\in G$$

(5c) $${\underline{Q}}_{G,i}\leq Q_{G,i}\leq {\bar{Q}}_{G,i},\;\;i\in G$$

where G is the set of generating units, including the reference bus generator, and the underlined and overlined symbols denote the lower and upper bounds.

#### 2.1.2. Transformer Constraints

The tap ratio of every regulating transformer is restricted to the interval selected for the test system:

(6) $${\underline{\tau }}_{i}\leq \tau_{i}\leq {\bar{\tau }}_{i},\;\;i\in T$$

Here, T is the set of tap-changing transformers.

#### 2.1.3. Shunt VAR Compensator Constraints

The reactive injection or absorption supplied by each shunt compensator must also remain within its prescribed range:

(7) $${\underline{Q}}_{sh,i}\leq Q_{sh,i}\leq {\bar{Q}}_{sh,i},\;\;i\in C_{sh}$$

where $C_{sh}$ identifies the set of shunt VAR compensation devices.

#### 2.1.4. Security Constraints

System security is enforced by bounding the voltage magnitudes at load buses and the apparent-power flow of each monitored branch:

(8) $${\underline{V}}_{PQ,i}\leq V_{PQ,i}\leq {\bar{V}}_{PQ,i},\;\;i\in L$$

(9) $$S_{br,l}\leq {\bar{S}}_{br,l},\;\;l\in B$$

In this notation, L is the load-bus set and B is the set of branches whose loading is constrained.

### 2.2. Objective Functions

During the MEBO-based optimization, each candidate solution is evaluated by four scalar indices: generation cost, fuel emission, real-power loss, and load-bus voltage deviation. The notation $f^{C}$, $f^{E}$, $f^{PL}$ and $f^{VD}$ is used below for these terms; they correspond to the same objective quantities used in the later weighted MO-OPF formulation.

#### 2.2.1. Quadratic Cost Function

The operating cost of the thermal units is represented with the conventional quadratic production cost-curve, written with revised coefficient notation as

(10) $$f^{C}=\sum_{i\in G}\left(A_{i}+B_{i}P_{G,i}+C_{i}P_{G,i}^{2}\right)$$

where $A_{i}$, $B_{i}$, and $C_{i}$ are the cost coefficients of generator i, and $P_{G,i}$ is its active-power output.

#### 2.2.2. Fuel Emission

Because electrical generation may produce atmospheric pollutants, the emission objective is included as an additional criterion. In this study, the emission index is minimized together with the other OPF objectives and is formulated as

(11) $$f^{E}=\sum_{i\in G}\left(\alpha_{i}+\beta_{i}P_{G,i}+\gamma_{i}P_{G,i}^{2}+\zeta_{i}exp\left(\rho_{i}P_{G,i}\right)\right)$$

where $f^{E}$, is expressed in ton/h, and $\alpha_{i}$, $\beta_{i}$, $\gamma_{i}$, $\zeta_{i}$, and $\rho_{i}$ are the emission coefficients of the *i*th generating unit.

#### 2.2.3. Transmission Real Power Losses

The network active-power loss is obtained from the difference between total generated active power and total active demand:

(12) $$f^{PL}=\sum_{i\in G}P_{G,i}-\sum_{j\in D}P_{D,j}$$

Here, D denotes the demand-bus set; therefore, the first summation covers generator injections and the second summation covers active load demands.

#### 2.2.4. Voltage Deviation

Keeping load-bus voltages close to their nominal values improves the voltage profile of the system [52]. The corresponding deviation index is computed as

(13) $$f^{VD}=\sum_{j\in D}\left|V_{j}-V^{ref}\right|,\;\;V^{ref}=1.0\;p.u.$$

where the summation is performed over all load buses. Thus, smaller values of $f^{VD}$ indicate a flatter and more desirable voltage profile.

## 3. Material and Method

**Figure 1:**
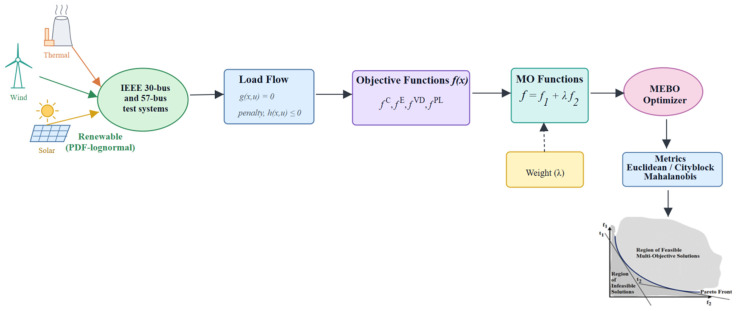
Overview of the system architecture.

When applying the proposed methodology to the test systems, system constraints make the overall framework highly complex, in addition to the uncertainty of renewable energy sources. The general architecture for solving this challenging problem is illustrated in Figure 1.

Figure 1 presents the general framework of the proposed MO-OPF methodology. In this study, the IEEE 30-bus and IEEE 57-bus test systems are considered as benchmark power systems, including conventional thermal generating units together with renewable energy sources such as wind and solar power plants. The network, generator, and load-flow data of the IEEE test systems are obtained from standard literature and previously published benchmark studies [21]. The renewable energy models are incorporated into the OPF formulation using probabilistic representations, where wind-speed and solar-radiation characteristics are modeled based on Weibull and lognormal probability density functions reported in the literature. The equality constraints are represented through the AC load-flow equations, while inequality constraints related to generator outputs, bus voltages, transformer tap settings, and transmission-line limits are handled through the penalty-based constraint mechanism shown in Figure 1. Four single-objective functions, namely, fuel cost ($f^{C}$), fuel emission ($f^{E}$), voltage deviation ($f^{VD}$), and active power loss ($f^{PL}$), are employed to formulate the multi-objective optimization problems. Based on these functions, several MO combinations, including cost-emission, cost–loss, cost–voltage deviation, emission–loss, thermal–renewable cost, and renewable source cost coordination, are investigated within the proposed MEBO-based optimization framework.

**Figure 2:**
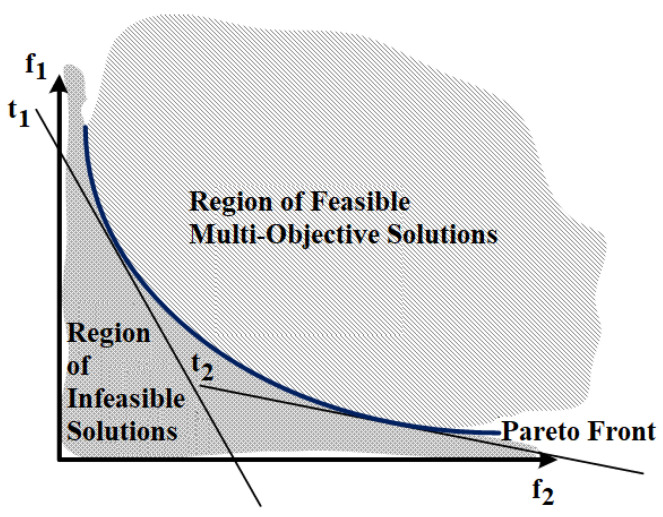
General theory of Pareto-Front for power flow problem.

In the study, the MO functions are constructed as the weighted sum of the single-objective functions where the weights are regarded as additional parameters that are subject to optimization. Single-objective functions have their own optimal solution set, but the optimal solution set for one single-objective function generally results in a non-optimal solution for another single-objective function. In order to find a compromise solution, the Pareto front, which contains the best points for one or more objective functions, is usually generated, and then a compromise point is selected depending on the trade-offs. Figure 2 shows a simple presentation of the Pareto theory where the Pareto front also separates feasible MO solutions from the infeasible solutions.

In the study, in order to find the best optimal solution set on the Pareto front, three different distance metrics are used, namely, Euclidean, Mahalanobis, and City Block, which are well known in the literature [53]. The Euclidean distance is the straight-line distance between two points. The Mahalanobis metric measures the distance between a point and a distribution. The square of the standard score represents the multivariate generalization of how many standard deviations the point deviates from the mean of the distribution. The City Block measures the distance as the sum of the absolute differences of their respective Cartesian coordinates. Using this metric, the length of the shortest grid path between any two spots is equal to the distance between them. The detailed mathematical formulas of the aforementioned distance metrics and their usage in the MO-OPF problem are given in the following section.

### 3.1. Proposed Methodology

The MO-OPF problem is addressed using the Modified EBO (MEBO) algorithm, a swarm optimization technique designed to optimize a single-objective function. Therefore, it is necessary to adapt the MEBO algorithm for the MO-OPF problem. The following subsection details the MEBO algorithm, while the subsequent section formulates the solution to the MO-OPF problem utilizing the MEBO optimizer. This explanation outlines how to tailor the MEBO algorithm for effectively solving the MO-OPF problem and achieving a compromised solution through distance metrics.

#### 3.1.1. Modified EBO Optimizer

**Figure 3:**
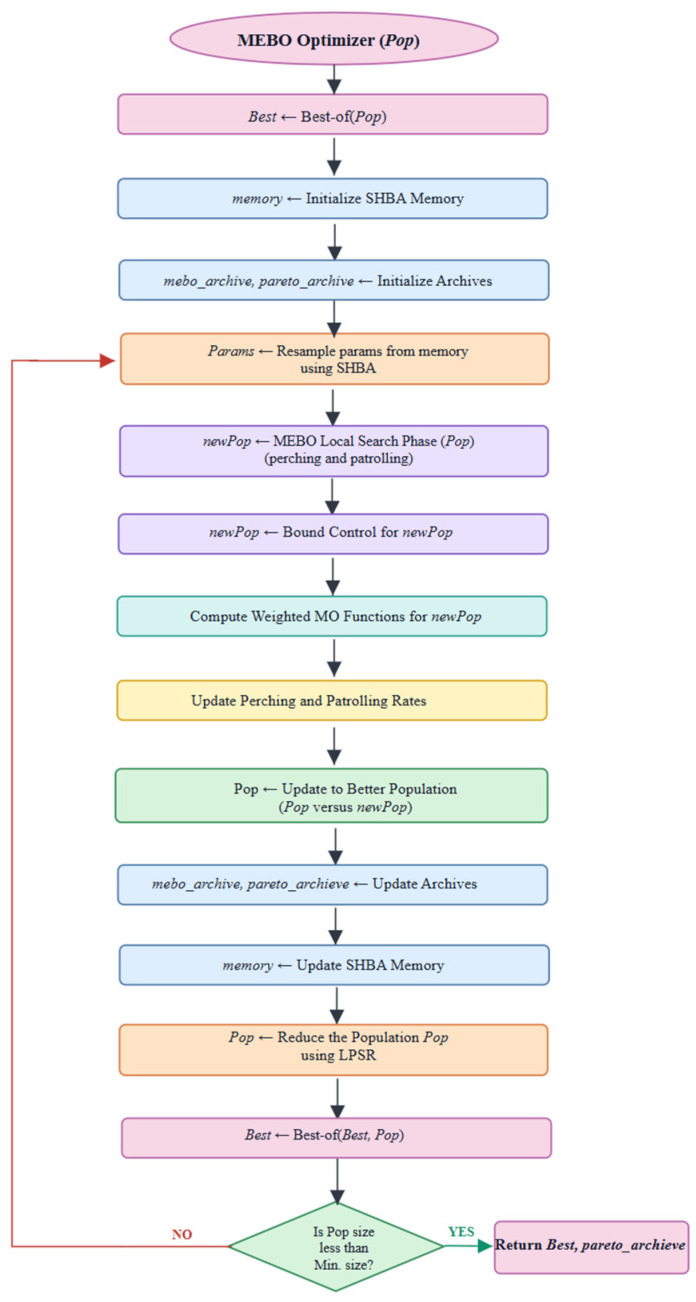
Modified EBO Phases for the MO-OPF Problem.

The Modified EBO algorithm here removes the CMAR (Covariance Matrix Adapted Retreat Phase) and SEQ (Sequential Quadratic Programming) phases from EBOwithCMAR, defined according to our implementation. The overall energy of EBOwithCMAR is distributed to these 3 phases to balance the exploration and exploitation of the algorithm. However, our motivation is to increase the exploration capability of the algorithm by relaxing the exploit focused phases such as SEQ. The flowchart of the Modified EBO algorithm is provided in general terms in Figure 3. The Modified EBO has three important features: i–Success History-Based Adaption (SHBA) to fine tune its internal parameters; ii–Linear Population Size Reduction (LPSR) to reduce the population size over time; and iii–History Achieve to keep the success history of previous solutions.

The MEBO is also modified to maintain an independent Pareto archive (*Pareto_archive*), in which the potential Pareto front points discovered by the MEBO are kept in the archive using non-dominance test across the population and archive. However, the pareto archive does not change the behavior of the MEBO; it just collects potential front points. MEBO has both internal and external parameters. The external parameters are *PSmin* and *PSmax* (minimum and maximum population sizes), *D* (dimension of the problem), *FEmax* (maximum number of function evaluations), *memSize* (memory size), and *archSize* (mebo archieve size). The *memory* can be considered as a table of internal parameters [*F*, *CR*, *T*, *Freq*] where the *memSize* defines the number of rows of the table. The default values of the internal parameters are given in Table 1 and subject to fine tuning over time. The memory is used for SHBA (success history-based adaptation) to fine tune the internal parameters, where *F* is the randomly sampled step size and *CR* is the crossover probability, which is randomly sampled to define the dimensions to update. Both *CR* and *F* are defined in terms of additional parameters *T* and *Freq*. These internal parameters mostly define the algorithm behavior in perching and patrolling phases of butterflies, which reflects the local searching.

The MEBO archive (*mebo_archive*) is another archive used to keep the success history of better solutions. It is initially empty and expanded with the improved solutions after the population update. The archive is primarily used in the local search phase to guide the current population. The Modified EBO has two local search equations, reflecting the perching and patrolling behavior of butterflies. The algorithm uses these two equations to generate a new solution from the current solution. The perching equation uses the archive to guide the current population.

The algorithm initializes the memory and archive with the default values, starting with the initial population containing uniform random solutions. The main loop of the algorithm first generates (resample) random values for internal parameters (*Params*) using SHBA and uses these values to guide the local searching of MEBO. The local searching phase generates an alternative population (*newPop*) by updating each solution using either a perching or patrolling equation, which is randomly determined based on perching and patrolling rates (*probPerch*, *probPat*). These rates are also internal parameters that are dynamically updated and fine-tuned. After the boundary control is ensured, the current population is updated to keep track of the improved solutions, when members of the current (*Pop*) versus the new population (*newPop*) are compared one by one. The newly improved (better) solutions are also added to the archive to maintain a success history. Then, using SHBA, each tuning parameter (e.g., *F*) in the memory is updated with the weighted average of all the particular param values associated with the improved solutions (success) in the current population. Further details of the parameter adaptation can also be found in [54,55]. Finally, the population size is reduced using LPSR if the number of function evaluation (*FE*) necessitates a reduction. According to LPSR, as the number FE calls gradually increases, the population size decreases at the same rate. Further details of MEBO can be found in [51].

#### 3.1.2. Adaptation of Modified EBO to Solve the MO-OPF Problem

**Figure 4:**
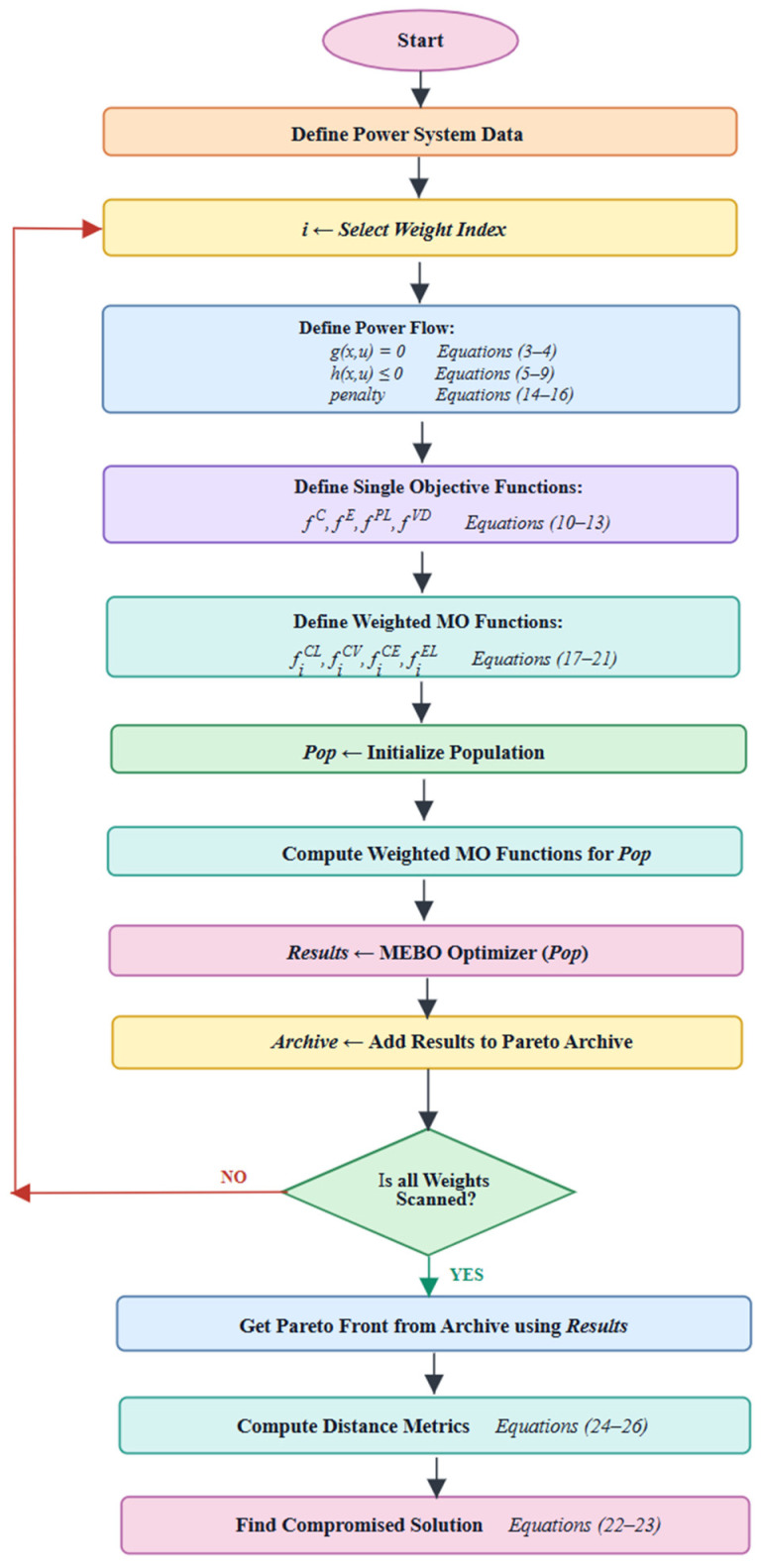
Adaptation of MEBO to solve the MO-OPF Problem.

In this study, MEBO is adapted to solve MO-OPF problems with bound constraints. The overall solution framework of the proposed approach is summarized in Figure 4. The flowchart shows all the phases from the system data preparation and objective function definition to finding a compromise solution.

First, the weight interval (*lower-upper* bounds) is divided into the exploit areas where the MEBO optimizer carries out a shallow search. MEBO maintains a Pareto archive, representing the front points during the search. Each exploit area is associated with an increasing weight $\lambda_{i}$, as shown in Figure 4. The function *select-weight-index* simply iterates from $\lambda_{min}$ to $\lambda_{max}$ where the number of weights ($\lambda_{i})$ decides the number of exploit regions. However, at the final iteration, when all the exploit regions are explored, except for the final one, the *select-weight-index* function computes the optimal weight index $i_{opt}$, formulated in Equation (22), to carry out a focused search on the optimal area. The population initialization at this iteration embeds the best solution associated with the optimum weight into the population, so that MEBO can focus on the optimal region.

The algorithm assumes bound constraints for the slack variable (P_Gslack_), bus load voltages (V_PQ_), and reactive power generations (Q_G_). Let the variable x denote any solution vector and Y = [V_PQ_, Q_G_] be the vector of constraint parameters, corresponding to the bus voltages and reactive power generations, respectively. Then, the objective function value, *f*(*x*), used at line-3 and line-20 of the algorithm, is updated by the sequential operations through (Equations (14)–(16)) to give the penalty to any violating bound. If the slack variable violates the bounds, it is penalized by weighting 1000, as shown in (Equation (15)).

(14) $$\left[P_{Glsack},Y]=PowerFlow(x\right)$$

(15) $$P_{Glsack}=1000*P_{Glsack}*(P_{Glsack}>{Ub}_{Glsack}||\;P_{Glsack}<{Lb}_{Glsack})$$

(16) $$Penalty\left(Y\right)=w*\sum_{i=1}^{n}\left[abs\left({Ub}_{i}-Y_{i}\right)*\left(Y_{i}>{Ub}_{i}\right)+abs\left({Lb}_{i}-Y_{i}\right)*\left(Y_{i}<{Lb}_{i}\right)\right]$$

where the PowerFlow(x) function is defined according to [56], ${Ub}_{Glsack}$ and ${Lb}_{Glsack}$ are the upper and lower bounds of the slack variable, respectively, ${Ub}_{i}$ and ${Lb}_{i}$ are the upper and lower bounds of the i. constraint parameters, respectively, and w is the penalty coefficient empirically found for each MO function definition. Then, the objective function value, f(x), used by the algorithm is computed using Equation (17).

(17) $$f(x)=\left\{\begin{array}{l} {f_{i}}^{CL}\left(x\right),\;if\;CL\\ {f_{i}}^{CV}\left(x\right),\;if\;CV\\ {f_{i}}^{CE}\left(x\right),if\;CE\\ {f_{i}}^{EL}\left(x\right),if\;EL \end{array}\right.$$

where the MO functions are defined on a $\lambda_{i}$ basis at Equations (18)–(21) for *CL* (cost–loss), *CV* (cost–voltage), *CE* (cost-emission), and *EL* (emission–loss), respectively.

(18) $${f_{i}}^{CL}\left(x\right)={f_{i}}^{C}\left(x\right)+\lambda_{i}*{f_{i}}^{L}\left(x\right)+Penalty\left(Y\right),\;\;with\;i=\{1\ldots k\}$$

(19) $${f_{i}}^{CV}\left(x\right)={f_{i}}^{C}\left(x\right)+\lambda_{i}*{f_{i}}^{V}\left(x\right)+Penalty\left(Y\right),\;\;with\;i=\{1\ldots k\}$$

(20) $${f_{i}}^{CE}\left(x\right)={f_{i}}^{C}\left(x\right)+\lambda_{i}*{f_{i}}^{E}\left(x\right)+Penalty\left(Y\right),\;\;with\;i=\{1\ldots k\}$$

(21) $${f_{i}}^{EL}\left(x\right)={f_{i}}^{E}\left(x\right)+\lambda_{i}*{f_{i}}^{L}\left(x\right)+Penalty\left(Y\right),\;\;with\;i=\{1\ldots k\}$$

where ${f_{i}}^{C}\left(x\right)$, ${f_{i}}^{L}\left(x\right),{f_{i}}^{V}\left(x\right),\;and\;{f_{i}}^{E}\left(x\right)$ are single objective function definitions for cost, loss, voltage deviation, and emission, respectively, for the current weight $\lambda_{i}$. The optimum $\lambda_{i}$ for each MO-OPF problem is found using sequential operations through Equations (22) and (23).

(22) $$i_{opt}=\underset{i=1\ldots k}{\mathrm{argmin}}({dist}_{M}(\vec{0},[{f_{i}}^{A},\;{f_{i}}^{B}]))$$

(23) $$compromised\;solution=x_{opt}=x_{i_{opt}}$$

where ${f_{i}}^{A}$ and ${f_{i}}^{B}$ are the converged objective values with the optimized solution $x_{i}$ associated with $\lambda_{i}$, the variable AB ϵ {CL, CV,CE,EL} and ${dist}_{M}$ is the distance function to compute the distance between two vectors according to metric M ϵ {Euclidean, cityblock, mahalanobis}. The distance metrics are formulized at Equations (24)–(26) for euclidean, cityblock, and mahalanobis, respectively.

(24) $${dist}_{euclid}\left(p,q\right)=\sqrt{\sum_{i=1\ldots k}{\left(p_{i}-q_{i}\right)}^{2}}$$

(25) $${dist}_{cityblock}\left(p,q\right)=\sum_{i=1\ldots k}\left|p_{i}-q_{i}\right|$$

(26) $${dist}_{mahalanobis}(p,\mu ,S)=\sqrt{{\left(p-\mu \right)}^{T}S^{-1}(p-\mu )}$$

where p and q are two vectors, μ is the mean, S is the covariance of a distribution, and mahalanobis distance is the distance of the vector p to the distribution.

## 4. Results and Discussions

In this paper, in order to indicate the efficiency and performance of the proposed compromised MO approach, two of the most widely used test systems, the IEEE 30 and 57-bus test systems, are employed. First, the IEEE 30 test system includes 30 buses, 41 branches, six generators, nine shunt capacitors, four on-load tap changers, 24 load buses, and 24 control and operating variables [57]. The minimum and maximum voltage values of the generator and load buses are 0.95 and 1.1 pu, respectively, whereas the minimum and maximum values of shunt capacitors are 0 and 0.05 pu, respectively. The IEEE 57-bus system was selected as the second test system. The system has an active power demand of 1250.8 MW and a reactive power demand of 336.4 MVAr. The fuel cost coefficients of the seven thermal generators located in the buses numbered 1, 2, 3, 6, 8, 9, and 12 were taken from reference number [21]. The minimum and maximum voltage values of the generator and load buses are 0.94 and 1.06 pu, respectively. In the test systems, the values of the on-load tap changers are expected to be between [0.95–1.1] and [0.90–1.1] with no minimum step size, respectively.

### 4.1. Classical Test System Studies

In this section, the MO functions given by Equations (18)–(21) are taken into consideration for the compromise optimization. For this purpose, the weight, namely λ, is chosen as the approximate ratio of the best single-objective solutions, which form the MO functions. The constraints of λ for each objective function are given in Table 2 below.

#### 4.1.1. Case 1 Fuel Cost and Fuel Emission Optimization

**Figure 5:**
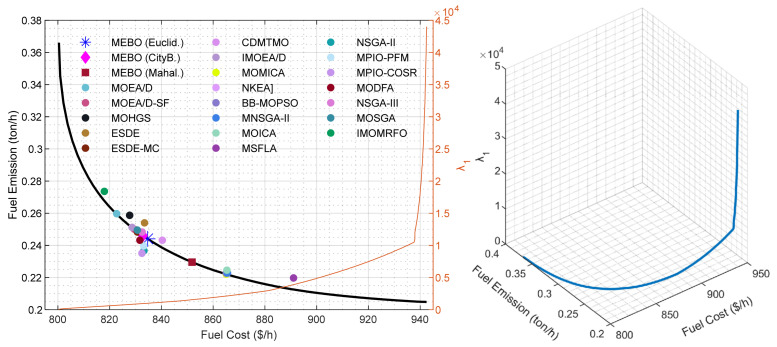
Convergence chart of compromise cost and emission optimization for the IEEE 30-bus system.

In this case, the MO function consisting of the fuel cost and fuel emission is employed for optimization. The convergence charts of the compromise MO solution, along with λ, are given in Figure 5. The euclidean, cityblock, and mahalanobis distances are obtained by 0.3426, 0.4838, and 4.0199, respectively. Also, the best compromise values for fuel cost and fuel emission achieved are 834.6765 $/h and 0.24406 ton/h, 832.8296 $/h and 0.24605 ton/h, and 851.8344 $/h and 0.22958 ton/h, respectively.

#### 4.1.2. Case 2 Fuel Cost and Power Loss Optimization

**Figure 6:**
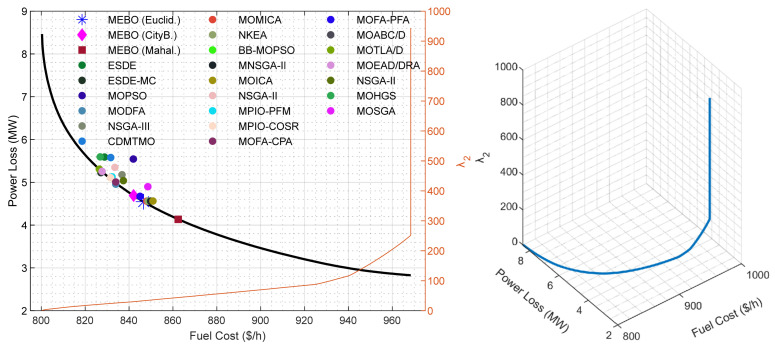
Convergence chart of compromised cost and power loss optimization.

The fuel cost and power loss MO optimization case is studied. The distances of euclidean, cityblock, and mahalanobis are obtained by 0.4111, 0.5791, and 4.7023, respectively. Also, the best compromise values of fuel cost and power loss achieved are: 846.6335 $/h and 4.5467 MW/h, 842.0758 $/h and 4.6910 MW/h, and 862.4056 $/h and 4.1357 MW/h, respectively. The convergence charts of the compromise MO solution are shown in Figure 6.

#### 4.1.3. Case 3 Fuel Cost and Voltage Deviation Optimization

**Figure 7:**
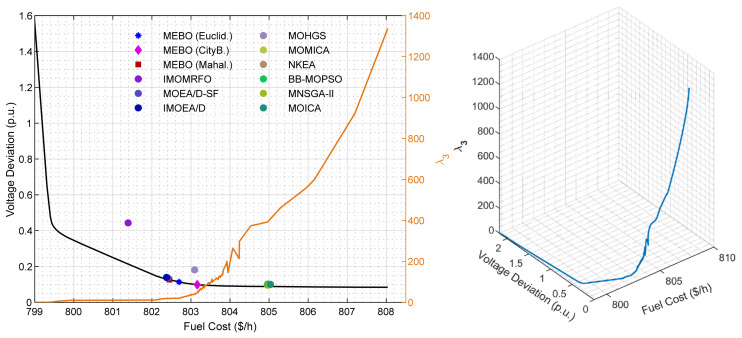
Convergence chart of compromise cost and voltage deviation optimization.

In this case, the MO function consisting of the fuel cost and voltage deviation is used for optimization. The convergence charts of the compromise MO solution, along with $\lambda_{3}$, are given in Figure 7. Also, the best compromise values for fuel cost and voltage deviation achieved are: 802.6948 $/h and 0.1139 pu, 803.1604 $/h and 0.0985 pu, and 802.4492 $/h and 0.1267 pu; the euclidean, cityblock, and mahalanobis distances are obtained by 0.0440, 0.0538, and 0.3752, respectively.

#### 4.1.4. Case 4 Fuel Emission and Power Loss Optimization

**Figure 8:**
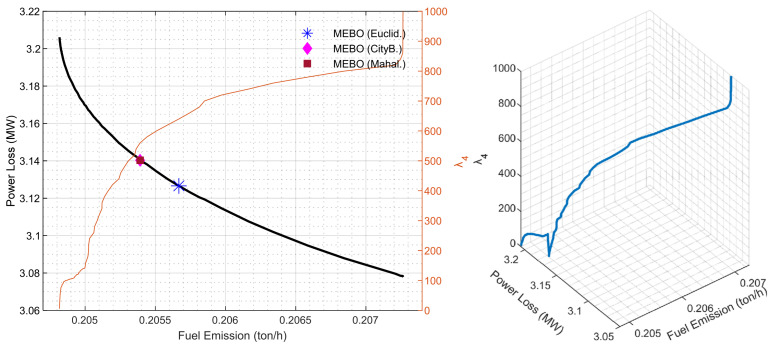
Convergence chart of compromise emission and power loss optimization.

MO optimization of fuel emission and power loss is studied and achieved. The distances of euclidean, cityblock, and mahalanobis are obtained by 0.5152, 0.7217, and 6.9277, respectively. The best compromise values of fuel emission and power loss are achieved as, 0.20567 ton/h and 3.1266 MW/h, 0.20539 ton/h and 3.1403 MW/h, and 0.20539 ton/h and 3.1403 MW/h, respectively. The convergence charts of the compromise MO solution are shown in Figure 8.

The distance metrics and Pareto optimization results are given in Table 3 below.

### 4.2. Comparison with the Literature for IEEE 30 Bus

In this study, three different metrics are employed to make decisions on the best compromise solutions for the IEEE 30 bus power system. In the literature, there are some studies consisting of Pareto optimal studies on the aforementioned power system. The comparison chart for the compromise MO optimization is given in Table 4 below. In our study, we sought to ensure transparency in the solutions obtained by applying the penalty technique across the entire solution space. To ensure a fair and transparent comparison, we included the limit values and dimensions (N) for the control variables used by the algorithms in the literature in the last column of the table. This column will help address concerns about whether the generated solutions were obtained under the same conditions. In the cost-emission optimization problem, the MEBO-Cityblock approach achieved a fuel cost of 832.8296 $/h together with 0.24605 ton/h emission, which is comparable to ESDE-MC [18] and NSGA-III [58], while providing a more balanced compromise between economic and environmental objectives. Compared with ESDE-MC [18], the proposed approach reduced the emission level from 0.2483 ton/h to 0.24605 ton/h while preserving a very close fuel-cost value. Similarly, although IMOMRFO [59] reported a lower fuel cost, the corresponding emission level increased significantly to 0.2736 ton/h, indicating a weaker compromise solution in terms of environmental performance. In addition, the MEBO-Mahalanobis approach achieved one of the lowest emission values among the compared methods with 0.22958 ton/h emission. For the cost–loss optimization problem, the proposed MEBO approaches also demonstrated strong performance. The MEBO-Euclidean solution achieved a 846.6335 $/h fuel cost with a transmission loss of 4.5467 MW, outperforming several recent methods such as MOSGA [3], MOFA-PFA [60], and BB-MOPSO [61] in terms of active power loss reduction. In particular, compared with MOSGA [3], the transmission loss was reduced from 4.8975 MW to 4.5467 MW, corresponding to an improvement of approximately 7.2%. Furthermore, the MEBO-Mahalanobis solution obtained the minimum transmission loss among all the compared approaches with 4.1357 MW, highlighting the strong exploitation capability of the proposed framework. In the cost–voltage deviation optimization problem, although some studies, such as MOEA/D-SF [21] and MOMICA [61], reported lower voltage deviation values, the proposed MEBO methods maintained competitive fuel-cost values together with stable and feasible operating conditions. In particular, the MEBO-Mahalanobis solution achieved a fuel cost of 808.9853 $/h while preserving acceptable voltage deviation characteristics. These results demonstrate that the proposed distance-based compromise mechanism can effectively balance conflicting objectives without significantly sacrificing economic performance. The proposed algorithm delivers outstanding solutions for the highly intricate and challenging fuel-cost and voltage-deviation MO-OPF problem. A close examination of Table 3 reveals that the compromise points for fuel cost and voltage deviation in Case 3 are 802.6948 $/h–0.1139 p.u., 803.1604 $/h–0.0985 p.u., and 802.4492 $/h–0.1267 p.u., respectively. When compared to the existing literature, these results represent the most favorable compromise outcomes to date. MEBO has outperformed widely recognized algorithms such as IMOEA/D [27], MOEA/D-SF [21], MOICA [58], MOHGS [24], MOMICA [58], IMOMRFO [59], BB-MOPSO [58], MNSGA-II [58], and MOICA [58]. For the emission–loss optimization case, the MEBO-Cityblock and MEBO-Mahalanobis approaches achieved an emission of 0.20539 ton/h with a 3.1403 MW power loss, indicating a highly balanced trade-off between environmental and operational objectives. Overall, the obtained results confirm that the proposed MEBO-based compromise strategy can successfully generate competitive Pareto solutions for different MO-OPF scenarios. Moreover, the utilization of different distance metrics provides additional flexibility for selecting the most suitable operating point according to the decision-maker’s priorities and system requirements.

### 4.3. Renewable Integrated Test System Studies

In this subsection on renewable plants, two wind farms and one PV system are integrated into the IEEE 30 bus test system in order to test the efficiency of the approach in the case of renewable plants. The wind farms are connected to buses 5 and 11 instead of thermal generators, while the PV system is integrated into bus 13 [21]. The wind power is predicted using the Weibull probability density function (PDF) in relation to the wind speed. The two decisive parameters of Weibull PDF are the scale and shape factor, denoted as c and k, respectively. Additionally, for the wind power estimation, the cut-in, rated, and cut-out speed parameters must be provided. Also, for the PV system, the generated power is heavily based on the irradiance value, whereas the PV power estimation is applied by using a lognormal PDF. To ensure a uniform system, the wind and PV parameters were taken from the literature [66]. Concerning wind power integration, overestimating power from an unpredictable source could lead to issues if the wind farm generates less energy than expected. In these circumstances, the system needs to run beyond its scheduled hours to guarantee an uninterrupted supply. The cost of allocating reserve generating units to wind farms in order to make up the overestimated amount is known as the reserve cost. Another unwanted scenario with wind farms in operation is when they generate more energy than expected. In these situations, the surplus electricity will be squandered if the operator complies with agreements and pays the penalty costs. The direct, reserve, and penalty cost functions of wind plants are given below, where $P_{ws,j}$, $P_{wav,j}$ and $f_{w}$ are the scheduled power, actual available power, and wind power probability, and $g_{j}$, $K_{Rw,j}$ and$,K_{Pw,j}$ direct, reserve, and penalty coefficients of the wind plant, which would be different depending on the characteristics of the plant.

(27) $$C_{w,j}\left(P_{ws,j}\right)=g_{j}P_{ws,j}$$

(28) $$\begin{array}{ll} C_{Rw,j}\left(P_{ws,j}-P_{wav,j}\right) & =K_{Rw,j}\left(P_{ws,j}-P_{wav,j}\right)\\ & =K_{Rw,j}\int_{0}^{P_{wsj}}\left(P_{ws,j}-p_{w,j}\right)f_{w}\left(p_{w,j}\right)dp_{w,j} \end{array}$$

(29) $$\begin{array}{ll} C_{P_{w},j}\left(P_{wav,j}-P_{ws,j}\right) & =K_{Pw,j}\left(P_{wav,j}-P_{ws,j}\right)\\ & =K_{Pw,j}\int_{P_{ws,j}}^{P_{w,j}}\left(p_{w,j}-P_{ws,j}\right)f_{w}\left(p_{w,j}\right)dp_{wj} \end{array}$$

The total cost of a wind plant is equal to sum of the direct, reserve, and penalty cost; $f_{cw}=\left(27\right)+\left(28\right)+(29)$.

PV sources also have different kinds of cost calculations in the literature, as given below. C_Rs_ is the reserve cost and C_Ps_ is the penalty cost, depending on the produced power and scheduled power.

(30) $$\begin{array}{rr} C_{Rs}\left(P_{ss}-P_{sav}\right) & =K_{Rs}.f_{s}\left(P_{sav}<P_{ss}\right).\left[P_{ss}-E\left(P_{sav}<P_{ss}\right)\right] \end{array}$$

(31) $$\begin{array}{rr} C_{Ps}\left(P_{sav}-P_{ss}\right) & =K_{Ps}.f_{s}\left(P_{sav}>P_{ss}\right).\left[E\left(P_{sav}>P_{ss}\right)-P_{ss}\right] \end{array}$$

where $K_{Rs}$ is the reserve cost coefficient, $K_{Ps}$ is the penalty cost coefficient, and $P_{sav}$ is the actual available power from the same plant. $f_{s}$ is the probability of a solar power shortage occurrence with respect to the scheduled power $P_{ss}$ for the reserve cost function, while it is the opposite for the penalty cost function. Also, E is the expectation of solar PV power below $P_{ss}$ for the reserve cost function, while it corresponds to PV solar power above the scheduled power for the penalty cost function. Thus, the total fuel cost can be defined as

(32) $$F_{total}={Cost}_{thermal}+{Cost}_{wind}+{Cost}_{PV}$$

The renewable wind parameters are chosen as 3 m/s for $v_{in}$, 16 m/s for $v_{r}$ and 25 m/s for $v_{out}$ for the wind systems connected to buses 5 and 11. Also, the rated power and the *c* and k parameters are chosen as 75 MW, 9, and 2 for wind system 1, while they are chosen as 60 MW, 10, and 2 for the wind system. The PV plant parameters are chosen as 50 MW rated power and 483 w/m^2^ for solar irradiance, while µ and *σ* are 6 and 0.6, respectively. By using the aforementioned wind and PV parameters, four different case studies are simulated for the renewable integrated test system.

The above stochastic representation is also consistent with recent studies on probabilistic and multi-objective OPF with renewable integration. In particular, ref. [33] models wind speed with a Weibull distribution and solar irradiance with a Beta distribution; ref. [41] represents wind, solar, and hydropower uncertainty by Weibull, lognormal, and Gumbel distributions, respectively; whereas [40] relies on forecasted wind-speed and solar-irradiance inputs in a hybrid wind/solar/thermal OPF setting.

#### 4.3.1. Case 5: Fuel Cost and Fuel Emission Optimization for Renewable Integrated Test Power System

**Figure 9:**
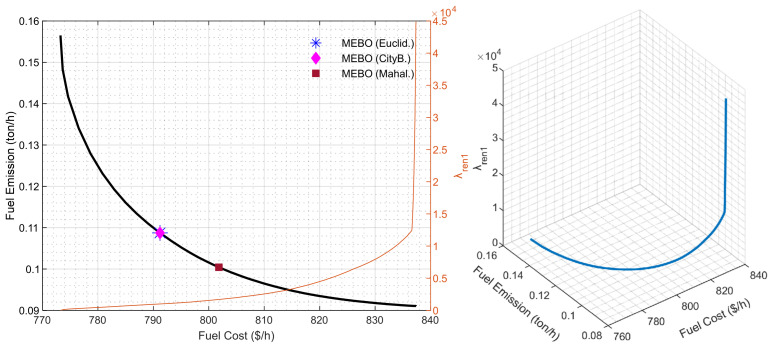
Convergence chart of compromise cost and emission Pareto optimization for renewable integrated power system.

In this case, the MO function, consisting of the fuel cost and fuel emission, is applied to a renewable-included power system. The convergence charts of the compromised MO solution, along with λ_ren1_, are given in Figure 9. The Euclidean, cityblock, and mahalanobis distances are obtained by 0.3888, 0.5498, and 6.6532, respectively. Also, the best compromise values of fuel cost and fuel emission are achieved as: 791.1951 $/h and 0.10873 ton/h, 791.1951 $/h and 0.10873 ton/h, 801.8172 $/h and 0.10044 ton/h, for Euclidean, cityblock, and mahalanobis-based Pareto optimization, respectively.

#### 4.3.2. Case 6 Fuel Cost and Power Loss Optimization for Renewable Integrated Test Power System

**Figure 10:**
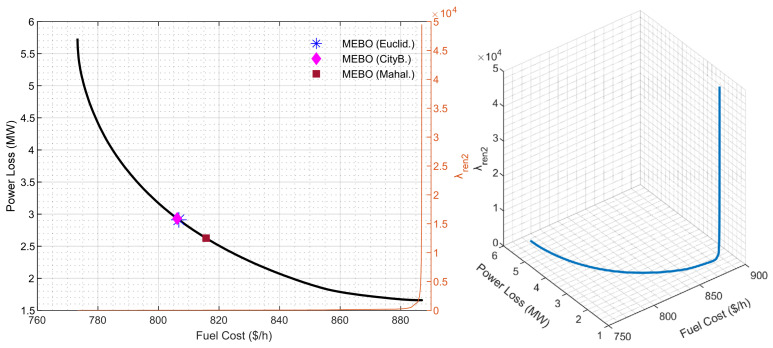
Convergence chart of compromised cost and power loss Pareto optimization for renewable integrated power system.

In this case, the MO function of fuel cost and power loss is achieved. The convergence charts of the compromise MO solution along with $\lambda_{ren2}$ are given in Figure 10. The Euclidean, cityblock, and mahalanobis distances are obtained by 0.4254, 0.6014, and 4.6503, respectively. Also, the best compromise values of fuel cost and power loss achieved are: 806.7230 $/h and 2.9117 MW, 806.1738 $/h and 2.9313 MW, 815.7048 $/h and 2.6263 MW, for Euclidean, cityblock, and mahalanobis-based Pareto optimization, respectively.

#### 4.3.3. Case 7 Thermal Fuel Cost and Renewable Cost Optimization for Renewable Integrated Test Power System

**Figure 11:**
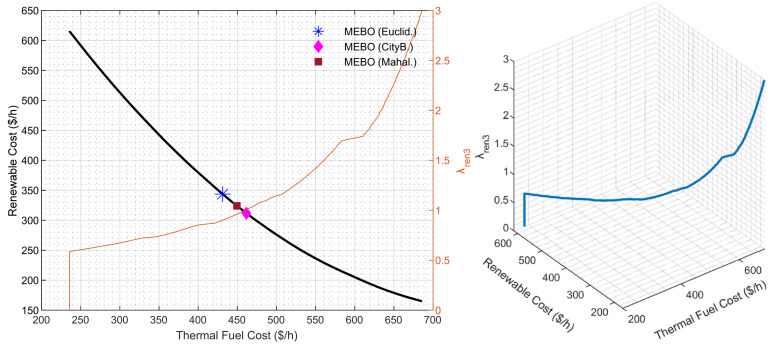
Convergence chart of compromise thermal cost and renewable cost Pareto optimization for a renewable integrated power system.

For this case, the thermal fuel cost and renewable cost are chosen as the target for MO optimization. The Euclidean, cityblock, and mahalanobis distances are obtained by 0.5882, 0.8274, and 13.7122 for this test case study, respectively. Also, the best compromise values of thermal fuel cost and renewable fuel cost achieved are: 431.2868 $/h and 343.5854 $/h, 461.5719 $/h and 311.6881 $/h, 449.4792 $/h and 324.0025 $/h, for euclidean, cityblock and mahalanobis-based Pareto optimization, respectively. The convergence characteristics of the Pareto optimization are given in Figure 11. These results indicate that the cityblock distance is the closest to the result of the single-objective fuel cost optimization given in (32), which is 773.2567 $/h.

#### 4.3.4. Case 8 Cost Optimization of Wind Turbine 1 and Wind Turbine 2 for a Renewable Integrated Test Power System

**Figure 12:**
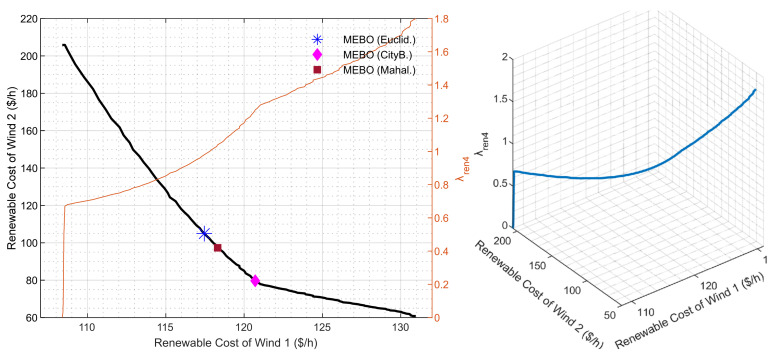
Convergence chart of compromise wind turbine 1 cost and wind turbine 2 cost Pareto optimization for renewable integrated power system.

In this case, the MO function of the renewable costs of wind turbines 1 and 2 for the corresponding busbars of 5 and 11 is achieved. The simulation scenario is adjusted so that the total fuel cost is closest to the original total fuel cost by optimizing the wind turbine costs. The convergence charts of the compromise MO solution along with $\lambda_{ren4}$ are given in Figure 12. The Euclidean, cityblock, and mahalanobis distances are obtained by 0.5040, 0.6759, and 6.5619, respectively. Also, the best compromise values of the wind turbine 1 cost and wind turbine 2 cost achieved are: 117.4559 $/h and 104.9287 $/h with a total fuel cost of 773.2843 $/h, 120.7073 $/h and 79.6548 $/h with a total fuel cost of 775.7280 $/h, and 118.3102 $/h and 97.3216 $/h with a total fuel cost of 773.3526 $/h, for euclidean, cityblock and mahalanobis-based Pareto optimization, respectively.

In this subsection, the proposed MEBO-based approach is tested under renewable integrated operating conditions through four different MO case studies, including fuel cost, emission, transmission loss, and renewable generation costs. The obtained results indicate that the proposed distance-based compromise strategy can produce feasible and competitive solutions for the IEEE 30-bus renewable integrated test system. In the cost-emission optimization problem, fuel cost values down to 791.1951 $/h are achieved, together with 0.10873 ton/h emission, while the Mahalanobis-based solution further reduces the emission value to 0.10044 ton/h. In the cost–loss optimization problem, the minimum transmission loss is reduced to 2.6263 MW, with acceptable fuel cost values. In addition, the renewable cost coordination studies show that the proposed approach can balance the renewable generation costs of wind and photovoltaic sources while keeping the total fuel cost close to the original operating condition. Overall, the Pareto-based results demonstrate that the proposed MEBO framework gives satisfactory results for renewable integrated MO-OPF problems under stochastic renewable generation conditions.

### 4.4. IEEE 57 Bus Test System Studies

In this section, we aimed to validate the performance of the MEBO-based weighting strategy we proposed, utilizing a larger test system. The outcomes of two case studies conducted on the IEEE 57-bus system, as outlined in the previous section, along with the relevant system parameters and explanations, are detailed in Table 5. This table presents the best results obtained after conducting 10 trials. In the following section, we will discuss three indicators—spread (Δ), hypervolume (HV), and Inverted Generational Distance (IGD)—to assess the statistical performance of the algorithm.

#### 4.4.1. Case 9 and 10 Cost–Emission and Cost–Loss Optimizations for 57 Bus Test System

**Figure 13:**
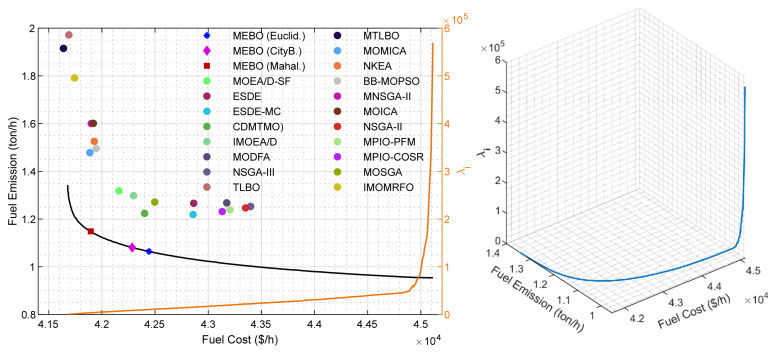
Convergence chart of compromise cost and emission optimization for the IEEE 57-bus system.

The first case study for the 57-bus test system focuses on the simultaneous optimization of fuel cost and fuel emission objective functions. The Pareto front for the compromise MO solution with $\lambda_{1}$ is shown in Figure 13. According to Table 5, the Euclidean, cityblock, and Mahalanobis distances are 0.3523, 0.4913, and 0.7480, respectively. The optimal values achieved for fuel cost and fuel emissions are as follows: for Euclidean optimization, the values are 42,443.0271 $/h and 1.0642 ton/h; for cityblock optimization, 42,285.0947 $/h and 1.0807 ton/h; and for Mahalanobis-based Pareto optimization, 41,895.1852 $/h and 1.1486 ton/h.

**Figure 14:**
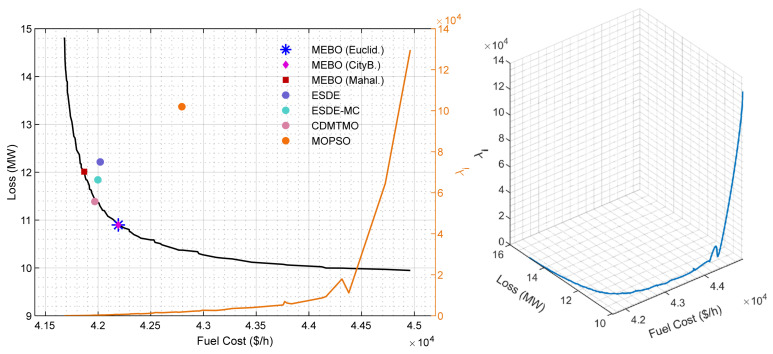
Convergence chart of compromise cost and loss optimization.

In Case 10, the optimization was performed on fuel cost and power loss objective functions. The convergence charts for the compromise MO solution, along with the $\lambda_{2}$, are shown in Figure 14. Here, the Euclidean, cityblock, and Mahalanobis distances are 0.2485, 0.3504, and 0.6853, respectively. The best optimized values achieved for fuel cost and power loss are: for Euclidean optimization, 42,192.0914 $/h and 10.8991 MW; for cityblock optimization, 42,192.0914 $/h and 10.8991 MW; and for Mahalanobis-based Pareto optimization, 41,868.7056 $/h and 12.0121 MW. Table 6 presents the results from various algorithms proposed in the literature for solving the same MO-OPF problem. The second column of this table provides the constraint value ranges for the test system used in these studies. This information is essential for ensuring that comparisons are conducted fairly and transparently. The results obtained in Case 9 are quite promising, outperforming nearly all algorithms evaluated under similar conditions. The compromise point of 42,443.0271 $/h achieved with the MEBO-Euclidean method outperformed algorithms such as IMOMRFO [59], MOMICA [58], NKEA [58], BB-MOPSO [58], MNSGA-II [58], MOICA [58], and MOEA/D-SF [21].

Additionally, it achieved a significantly lower emission value of 1.0642 ton/h, marking a major success. Though MTLBO [20] recorded the lowest cost at 41,638.38 $/h, it also resulted in higher emissions of 1.9152 ton/h. In Case 10, both the MEBO-Euclidean and MEBO City Block algorithms achieved a compromise point at 42,192.0914 $/h and 10.8991 MW, respectively. The power loss value attained in both distance metrics is the lowest reported in the literature. However, while the algorithm reduced this loss value, it made a relative compromise on the cost. In Table 6, it is noted that the CDMTMO [60] algorithm, under the same conditions, produced a lower cost of 41,968.88347 $/h than MEBO but increased the power loss to 11.388 MW. The ESDE-MC [18] algorithm also achieved a favorable solution on the cost side, with 41,998.3588 $/h and 11.8415 MW, though it is important to note that the shunt VAR limit for this algorithm is set to 0–0.03 p.u.

#### 4.4.2. Statistical Performance

In this section, we evaluate the quality of the Pareto curves that make up the solution set of the MEBO-based optimization algorithm utilizing the weighting method, employing the spread (Δ), hypervolume (HV) and Inverted Generational Distance (IGD) test indices [67]. A high value of the spread metric (Δ), which assesses the even distribution of solutions along the Pareto curve and encompasses the endpoints, suggests an irregular distribution. The calculation is performed as follows.

(33) $$∆=\frac{\sum_{i=1}^{k}d\left(E_{i},Ω\right)+\sum_{X\in Ω}\left|d\left(X,Ω\right)-d_{avg}\right|}{\sum_{i=1}^{k}d\left(E_{i},Ω\right)+(\left|Ω\right|-k)d_{avg}}$$

Here, Ω denotes the set of non-dominated solutions, $E_{i}$ represents the distance of the i-th solution from the true Pareto front, and k denotes the target number. The minimum distance d and the average distance $d_{avg}$ between two solutions are calculated as follows:

(34) $$d(X,Ω)=\underset{Y\in Ω,Y\neq X}{\min}\left\|F\left(X\right)-F(Y)\right\|$$

(35) $$d_{avg}=\frac{1}{\left|Ω\right|}\sum_{X\in Ω}d(X,Y)$$

The HV metric, which measures the multidimensional volume the Pareto curve covers relative to a reference point, ranges from 0 to 1, with higher values indicating better performance. HV is calculated as follows.

(36) $$HV=\overset{\left|Ω\right|}{\underset{i=1}{⋃}}v_{i}$$

where $v_{i}$ is the hypercube in the target space between the i-th solution and the reference point.

The IGD metric, which measures the average distance from each reference point in the true Pareto front to the nearest solution in the approximation set, ranges from 0 to ∞, with lower values indicating better convergence and distribution performance. IGD is calculated as follows.

(37) $$IGD=\frac{1}{\left|P*\right|}\sum_{i=1}^{\left|P*\right|}d(v_{i},\;A)$$

where d(vi, A) is the minimum Euclidean distance from the i-th point vi in the reference Pareto front P∗ to its closest solution in the approximation set A, and |P∗| is the total number of reference points in P∗.

The MO-OPF problem is inherently complex and presents significant challenges. We endeavored to address this intricate issue using the MEBO algorithm, recognized as one of the advanced approaches, along with the weighting methodology we proposed. The effectiveness of this approach was assessed through two key metrics, namely, spread and hypervolume, in multi-objective Pareto-based optimization problems, utilizing a large-scale test system with 57 buses. The results from these metrics underwent the IGD test, which confirmed that our algorithm demonstrates low dispersion and achieves a high level of convergence accuracy.

**Figure 15:**
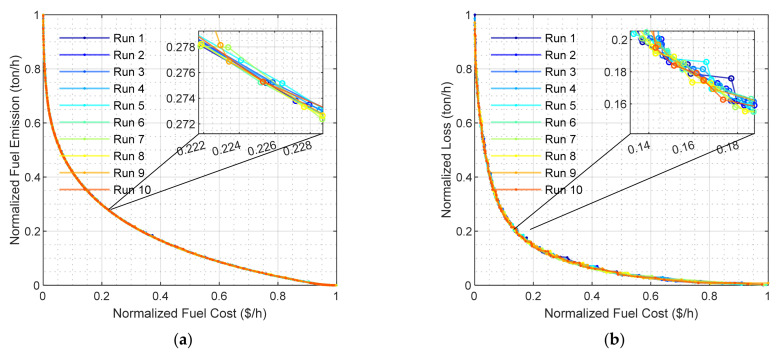
Running 10 times run for Pareto fronts (**a**) Case 9 (**b**) Case 10.

The MO-OPF problem is inherently complex and presents significant challenges. We endeavored to tackle this difficult issue, as illustrated in Figure 15, which displays the Pareto curves obtained for Case 9 and Case 10 after ten repetitions. To effectively differentiate the curves, it was necessary to employ a broader window. This observation also confirms that the algorithm exhibits low variance. The standard deviation values for each case are provided in Table 7. The low values underscore the importance of Figure 15a,b.

**Figure 16:**
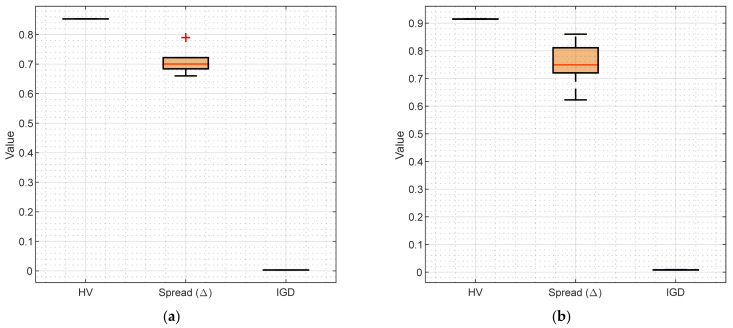
Ten runs for box plots: (**a**) Case 9 (**b**) Case 10. In each box plot, the central red line indicates the median, the lower and upper edges of the box denote the 25th and 75th percentiles, the black whiskers extend to the most extreme data points not considered outliers, and the red ‘+’ markers represent the outliers.

**Figure 17:**
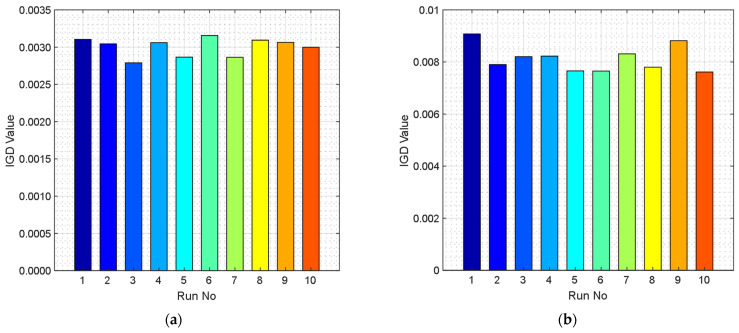
Ten runs for IGD test results: (**a**) Case 9 (**b**) Case 10.

The box plots presented in Figure 16a,b clearly indicate that the HV values for both scenarios are very close to 1. Additionally, it is noteworthy that the Δ values are quite small. It is essential to subject the Δ and HV indicators to the IGD test, the results of which are depicted in Figure 17a,b. In Case 9, where optimization focused on fuel cost and fuel emission objective functions, the average IGD value was recorded at 0.003. This outcome confirms that the solutions generated are in close proximity to the Pareto front. Conversely, in Case 10, which involved the optimization of fuel cost and power loss, the average IGD value was slightly higher, at 0.0081. This increase in the IGD value in comparison to Case 9 can be attributed to the greater complexity of the problem. The relatively low standard deviations for both cases further highlight that the proposed MEBO-based weighting strategy approach yields stable results, producing high-quality solutions that are near the true Pareto front within the solution space of the MO-OPF problem.

## 5. Conclusions

This study presents an adaptation of Modified Effective Bufferfly Optimization in solving the multi-objective optimal power flow problem under tight constraints. The main focus of the study is on proposing a robust method to find a compromise solution, rather than exploring the full Pareto front curve. The suggested method uses multiple shallow searches, each associated with a weight and Pareto archive, before carrying out a deep search of the region identified with the optimal weight to focus on compromise solutions. This research examines the efficiency of the proposed method under optimal scenarios for the control variables using the conventional IEEE 30-bus and 57-bus power systems with the multiple objectives of cost–emission, cost–loss, cost–voltage deviation, emission–loss, thermal–renewable cost, and renewable source cost coordination, considering fuel cost, fuel emission, power loss, and voltage deviation. Additionally, by including renewable plants, it is shown that not only can thermal fuel cost and system variables be optimized, but renewable plant power values may also be chosen optimally for different scenarios. Compromise optimal values are successfully achieved. The most secure and ideal method of operating the system under stress was found to be the classical linear approach to MO optimization techniques. With our findings, it is also possible to compare the results with other state-of-the-art literature research or with results from the same study by utilizing distinct distance measurements.

In this study, three different metrics are employed to make decisions on the optimal compromise solutions for a large-scale IEEE 57-bus power system. In the literature, there are some works consisting of Pareto optimal studies on the aforementioned power system. It is seen that, for Case 1, the proposed MEBO optimization with the cityblock distance metric produces better results than MOMICA, NKEA, BB-MOPSO, MNSGA-II, and MOICA with respect to fuel cost. Also, the proposed approach is better than MOPSO, MOFA-CPA and MOFA-PFA for compromise optimization solutions. For Case 2, the proposed approach of MEBO-Cityblock outperforms the MOMICA, NKEA, BB-MOPSO, MNSGA-II, and MOICA, while the MEBO-Euclidean approach is better than MOTLA/D and MOEAD/DRA in terms of real power loss. Moreover, all the MEBO approaches produce better compromise solution results than MOEA/D, MOPSO, and NSGA-II for voltage deviation.

In this paper, the key findings are as follow:

- In case 1, the best compromise values of fuel cost and fuel emission achieved are: 834.6765 $/h and 0.24406 ton/h, 832.8296 $/h and 0.24605 ton/h, 851.8344 $/h and 0.22958 ton/h, respectively.
- In case 2, the best compromise values of fuel cost and power loss achieved are: 846.6335 $/h and 4.5467 MW/h, 842.0758 $/h and 4.6910 MW/h, 862.4056 $/h and 4.1357 MW/h, respectively.
- In case 3, the best compromise values of fuel cost and voltage deviation achieved are: 812.6559 $/h and 0.27381 pu, 812.6559 $/h and 0.27381 pu, 808.9853 $/h and 0.33774 pu, respectively.
- In case 4, the best compromise values of fuel emission and power loss achieved are: 0.20567 ton/h and 3.1266 MW/h, 0.20539 ton/h and 3.1403 MW/h, 0.20539 ton/h and 3.1403 MW/h, respectively.
- In case 5, the best compromised values of fuel cost and fuel emission are achieved as; 791.1951 $/h and 0.10873 ton/h, 791.1951 $/h and 0.10873 ton/h, 801.8172 $/h and 0.10044 ton/h, for euclidean, cityblock and mahalanobis-based Pareto optimization, respectively.
- In case 6, the best compromise values of fuel cost and power loss achieved are: 806.7230 $/h and 2.9117 MW, 806.1738 $/h and 2.9313 MW, 815.7048 $/h and 2.6263 MW, for euclidean, cityblock, and mahalanobis-based Pareto optimization, respectively.
- In case 7, the best compromise values of thermal fuel cost and renewable fuel cost achieved are: 431.2868 $/h and 343.5854 $/h, 461.5719 $/h and 311.6881 $/h, 449.4792 $/h and 324.0025 $/h, for euclidean, cityblock, and mahalanobis-based Pareto optimization, respectively.
- In Case 8, the costs of wind turbine 1 and wind turbine 2 were optimized simultaneously while keeping the total fuel cost close to its original value; to avoid repetition, the corresponding numerical values are reported only in the Results and Discussion section and are not restated here.
- In case 9, the best compromise values of wind turbine 1 cost and wind turbine 2 cost achieved are: 117.4559 $/h and 104.9287 $/h with a total fuel cost of 773.2843 $/h, 120.7073 $/h and 79.6548 $/h with a total fuel cost of 775.7280 $/h, 118.3102 $/h and 97.3216 $/h with a total fuel cost of 773.3526 $/h, for euclidean, cityblock, and mahalanobis-based Pareto optimization, respectively.
- In case 9, the best compromise values of fuel cost and fuel emission achieved are: 42,443.0271 $/h and 1.0642 ton/h, 42,285.0947 $/h and 1.0807 ton/h, 41,895.1852 $/h and 1.1486 ton/h, for Euclidean, cityblock, and mahalanobis-based Pareto optimization, respectively.
- In case 10, the best compromise values of fuel cost and fuel emission achieved are: 42,192.0914 $/h and 10.8991 MW, 42,192.0914 $/h and 10.8991 MW, 41,868.7056 $/h and 12.0121 MW, for Euclidean, cityblock, and mahalanobis-based Pareto optimization, respectively.

In future studies, we plan to verify the proposed approach in real operating conditions to prove its robustness.

## Figures and Tables

**Table 1:** The parameter values of the Modified EBO.

Parameter/Symbol	Value or Setting Used
*PSmax*, *PSmin*, *PS*	*PSmax* = 18 × *D* → *PSmin* = 4
*memSize*	6
*F*	0.7
*CR*	0.5
*T*	0.1
*Freq*	0.5
*archSize*	2.6 × *PS*
*FEmax*	25,000
*probPerch*, *probPat*	Initial 0.5, 0.5; adaptive bounds (0–1)

**Table 2:** Constraints of λ.

MO Function	Minimum	Maximum
*f^CE^*	0	44.01 × 10^3^
*f^CL^*	0	1 × 10^3^
*f^CV^*	0	15 × 10^3^
*f^EL^*	0	1 × 10^3^

**Table 3:** Distances and Corresponding Values of Cases 1–4.

**Metric**	**Case 1**			**Case 2**		
	**Dist.**	$f^{C}$ **($/h)**	$f^{E}$ **(ton/h)**	**Dist.**	$f^{C}$ **($/h)**	$f^{PL}$ **(MW)**
Euclidean	0.3426	834.6765	0.24406	0.4111	846.633	4.5467
Cityblock	0.4838	832.8296	0.24605	0.5791	842.075	4.6910
Mahalanobis	4.0199	851.8344	0.22958	4.7023	862.405	4.1357
**Metric**	**Case 3**			**Case 4**		
	**Dist.**	$f^{C}$ **($/h)**	$f^{VD}$ **(pu)**	**Dist.**	$f^{E}$ **(ton/h)**	$f^{PL}$ **(MW)**
Euclidean	0.0440	802.6948	0.1139	0.515	0.2056	3.1266
Cityblock	0.0538	803.1604	0.0985	0.721	0.2053	3.1403
Mahalanobis	0.3752	802.4492	0.1267	6.927	0.2053	3.1403

**Table 4:** Comparison of simulation results with the literature for Cases 1–4.

Case No	Methods	Existing Methods Variables Limits		$f^{C}$	$f^{E}$	$f^{PL}$	$f^{VD}$
		$\left[{\underline{V}}_{G}-{\bar{V}}_{G}],[\underline{\tau }-\bar{\tau }],[{\underline{Q}}_{sh}-{\bar{Q}}_{sh}],[{\underline{V}}_{PQ}-{\bar{V}}_{PQ}\right]$	N				
**Case 1**	MOEA/D [21]	[0.95–1.1], [0.9–1.1], [0–0.05], [0.95–1.05]	24	822.716	0.25970	-	-
	MOEA/D-SF [21]			829.515	0.25011	-	-
	MOHGS [24]	[0.95–1.1], [0.9–1.1], [0–0.05], [0.95–1.05]	24	827.735	0.2587	-	-
	ESDE [18]	[0.9–1.1], [0.9–1.1], [0–0.05], [0.9–1.1]	24	833.474	0.2540	-	-
	ESDE-MC [18]			830.718	0.2483	-	-
	CDMTMO [60]	[0.95–1.1], [0.9–1.1], [0–0.05], [0.95–1.05]	24	840.3490	0.2432	-	-
	IMOEA/D [27]	Not Reported		828.674	0.2512	-	-
	MOMICA [58]	[0.95–1.1], [0.9–1.1], [0–0.05], [0.95–1.05]	24	865.066	0.2221	-	-
	NKEA [58]			865.0815	0.2228	-	-
	BB-MOPSO [58]			865.0985	0.2227	-	-
	MNSGA-II [58]			865.5264	0.2228	-	-
	MOICA [58]			865.3184	0.2246	-	-
	MSFLA [17]	[0.95–1.05], [0.9–1.05], [0–0.2], [0.95–1.05]	17	891.06507	0.21973	-	-
	NSGA-II [61]	[0.95–1.1], [0.9–1.1], [0–0.05], [0.95–1.05]	24	833.2605	0.2367	-	-
	MPIO-PFM [61]			833.1703	0.2397	-	-
	MPIO-COSR [61]			832.4655	0.2351	-	-
	MODFA [62]	[0.95–1.1], [0.9–1.1], [0–0.05], [0.95–1.1]	24	831.6652	0.2432	-	-
	NSGA-III [62]			832.5323	0.2483	-	-
	MOSGA [3]	[0.95–1.1], [0.9–1.1], [0–0.05], [0.95–1.1]	24	830.6940	0.2495	-	-
	IMOMRFO [59]	[0.95–1.1], [0.9–1.1], [0–0.05], [0.95–1.05]	24	817.9615	0.2736		-
	**MEBO-Euclidean**	[0.95–1.1], [0.9–1.1], [0–0.05], [0.95–1.1]	24	**834.6765**	**0.24406**	**-**	-
	**MEBO-Cityblock**			**832.8296**	**0.24605**	**-**	**-**
	**MEBO-Mahalanobis**			**851.8344**	**0.22958**	**-**	**-**
**Case 2**	ESDE [18]	[0.9–1.1], [0.9–1.1], [0–0.05], [0.9–1.1]	(24) -	828.8413	-	5.5901	-
	ESDE-MC [18]			827.1592	-	5.2270	-
	MOPSO [63]	[0.95–1.05], [0.9–1.05], [0–0.2], [0.95–1.05]	17	841.9512	-	5.5436	-
	MODFA [62]	[0.95–1.1], [0.9–1.1], [0–0.05], [0.95–1.1]	24	833.9365	-	4.9561	-
	NSGA-III [62]			836.8076	-	5.1775	-
	CDMTMO [60]	[0.95–1.1], [0.9–1.1], [0–0.05], [0.95–1.05]	17	831.6472	-	5.5788	-
	MOMICA [58]	[0.95–1.1], [0.9–1.1], [0–0.05], [0.95–1.05]	24	848.0544	-	4.5603	-
	NKEA [58]			848.68	-	4.5615	-
	BB-MOPSO [58]			849.7496	-	4.5607	-
	MNSGA-II [58]			849.8742	-	4.5608	-
	MOICA [58]			850.9001	-	4.5625	-
	NSGA-II [61]	[0.95–1.1], [0.9–1.1], [0–0.05], [0.95–1.05]	24	833.5363	-	5.3483	-
	MPIO-PFM [61]			832.2274	-	5.1270	-
	MPIO-COSR [61]			831.5576	-	5.1085	-
	MOFA-CPA [64]	[0.95–1.1], [0.9–1.1], [0–0.05], [0.95–1.1]	24	833.94	-	5.0075	-
	MOFA-PFA [64]			845.01	-	4.6727	-
	MOABC/D [65]	[0.95–1.1], [0.9–1.1], [0–0.05], [Not Available]	24	827.636	-	5.2451	-
	MOTLA/D [65]			826.446	-	5.3074	-
	MOEAD/DRA [65]			827.717	-	5.2556	-
	NSGA-II [65]			837.416	-	5.0397	-
	MOHGS [24]	[0.95–1.1], [0.9–1.1], [0–0.05], [0.95–1.05]	24	826.842	-	5.5946	-
	MOSGA [3]	[0.95–1.1], [0.9–1.1], [0–0.05], [0.95–1.05]	24	848.5596	-	4.8975	-
	**MEBO-Euclidean**	[0.95–1.1], [0.9–1.1], [0–0.05], [0.95–1.1]	24	**846.6335**	**-**	**4.5467**	**-**
	**MEBO-Cityblock**			**842.0758**	**-**	**4.6910**	**-**
	**MEBO-Mahalanobis**			**862.4056**	**-**	**4.1357**	
**Case 3**	IMOMRFO [59]	[0.95–1.1], [0.9–1.1], [0–0.05], [0.95–1.05]	24	801.3908	-	-	0.4435
	MOEA/D-SF [21]	[0.95–1.1], [0.9–1.1], [0–0.05], [0.95–1.05]	24	802.406	-	-	0.136
	IMOEA/D [27]	NR	-	802.374	-	-	0.139
	MOHGS [24]	[0.95–1.1], [0.9–1.1], [0–0.05], [0.95–1.05]	24	803.094	-	-	0.181
	MOMICA [58]	[0.95–1.1], [0.9–1.1], [0–0.05], [0.95–1.05]	24	804.9611	-	-	0.095
	NKEA [58]			804.9612	-	-	0.099
	BB-MOPSO [58]			804.9639	-	-	0.102
	MNSGA-II [58]			805.0076	-	-	0.098
	MOICA [58]			805.0345	-	-	0.100
	**MEBO-Euclidean**	[0.95–1.1], [0.9–1.1], [0–0.05], [0.95–1.1]	24	**802.6948**	-	-	**0.1139**
	**MEBO-Cityblock**			**803.1604**	-	-	**0.0985**
	**MEBO-Mahalanobis**			**802.4492**	-	-	**0.1267**
**Case 4**	**MEBO-Euclidean**	[0.95–1.1], [0.9–1.1], [0–0.05], [0.95–1.1]	24	**-**	**0.20567**	**3.1266**	**-**
	**MEBO-Cityblock**			**-**	**0.20539**	**3.1403**	**-**
	**MEBO-Mahalanobis**			**-**	**0.20539**	**3.1403**	**-**

**Table 5:** Distances and Corresponding Values of Cases 9–10.

Metric	Case 9			Case 10		
	Dist.	$f^{C}$ ($/h)	$f^{E}$ (ton/h)	Dist.	$f_{C}$ ($/h)	$f^{PL}$ (MW)
Euclidean	0.2485	42,192.0914	10.8991	0.3523	42,443.0271	1.0642
Cityblock	0.3504	42,192.0914	10.8991	0.4913	42,285.0947	1.0807
Mahalanobis	0.6853	41,868.7056	12.0121	0.7480	41,895.1852	1.1486

**Table 6:** Comparison of simulation results with the literature for Cases 9–10.

Case No	Methods	Existing Methods Variables Limits		$f_{C}$	$f_{E}$	$f_{PL}$
		$\left[{\underline{V}}_{G}-{\bar{V}}_{G}],[\underline{\tau }-\bar{\tau }],[{\underline{Q}}_{sh}-{\bar{Q}}_{sh}],[{\underline{V}}_{PQ}-{\bar{V}}_{PQ}\right]$	N			
**Case 9**	MOEA/D-SF [21]	[0.95–1.1], [0.9–1.1], [0–0.2], [0.94–1.06]	33	42,160.09	1.3183	-
	ESDE [18]	[0.9–1.1], [0.9–1.1], [0–0.3], [0.94–1.06]	33	42,863.3243	1.2662	-
	ESDE-MC [18]			42,857.4869	1.2191	-
	CDMTMO [60]	[0.95–1.1], [0.9–1.1], [0–0.2], [0.94–1.06]	33	42,401.9457	1.22387	-
	IMOEA/D [27]	NR		42,297.43	1.298	-
	MODFA [62]	[0.9–1.1], [0.9–1.1], [0–0.3], [0.9–1.1]	33	43,174.5740	1.2679	-
	NSGA-III [62]			43,398.7547	1.2530	-
	TLBO [20]	[0.95–1.05], [0.9–1.1], [0–0.3], [NR]	33	41,688.74	1.9716	-
	MTLBO [20]			41,638.38	1.9152	-
	MOMICA [58]	[0.9–1.1], [0.9–1.1], [0–0.3], [0.94–1.06]	33	41,886.7982	1.4784	-
	NKEA [58]			41,928.8054	1.5256	-
	BB-MOPSO [58]			41,947.3505	1.4957	-
	MNSGA-II [58]			41,899.9527	1.6004	-
	MOICA [58]			41,919.7061	1.601	-
	NSGA-II [61]	[0.9–1.1], [0.9–1.1], [0–0.3], [0.9–1.1]	33	43,351.1353	1.2466	-
	MPIO-PFM [61]			43,205.8477	1.2386	-
	MPIO-COSR [61]			43,131.2743	1.2314	-
	MOSGA [3]	[0.95–1.1], [0.9–1.1], [0–0.02], [0.94–1.06]	33	42,497.013	1.2712	-
	IMOMRFO [59]	[0.95–1.1], [0.9–1.1], [0–0.02], [0.94–1.06]	33	41,742.9442	1.7912	-
	**MEBO-Euclidean**	[0.95–1.1], [0.9–1.1], [0–0.2], [0.94–1.06]	33	**42** **,** **443.0271**	**1.0642**	-
	**MEBO-Cityblock**			**42** **,** **285.0947**	**1.0807**	-
	**MEBO-Mahalanobis**			**41** **,** **895.1852**	**1.1486**	-
**Case 10**	ESDE [18]	[0.9–1.1], [0.9–1.1], [0–0.3], [0.94–1.06]	33	42,020.7439	-	12.2155
	ESDE-MC [18]			41,998.3588	-	11.8415
	CDMTMO [60]	[0.95–1.1], [0.9–1.1], [0–0.2], [0.94–1.06]	33	41,968.88347	-	11.388
	MOPSO [63]	[0.95–1.1], [0.9–1.1], [0–0.2], [0.94–1.06]	33	42,795	-	13.37
	**MEBO-Euclidean**	[0.95–1.1], [0.9–1.1], [0–0.2], [0.94–1.06]	**33**	**42** **,** **192.0914**	**-**	**10.8991**
	**MEBO-Cityblock**			**42** **,** **192.0914**	**-**	**10.8991**
	**MEBO-Mahalanobis**			**41** **,** **868.7056**	**-**	**12.0121**

NR (Not Reported).

**Table 7:** Statistical results for Case 9–10 after 10 runs.

Case Study	Δ		HV		IGD	
	Avg.	Std.	Avg.	Std.	Avg.	Std.
**Case 9**	0.7047	0.0381	0.8531	0.0001	0.0030	0.0001
**Case 10**	0.7499	0.0739	0.9148	0.0005	0.0081	0.0005

## Data Availability

The benchmark IEEE 30-bus and IEEE 57-bus system data and renewable energy parameters used in this study are available from the published sources cited in the manuscript. The numerical results generated and analyzed during this study are included in this article. Further details are available from the corresponding author upon reasonable request.

## Abbreviations

**Abbreviation/Symbol**	**Definition**	**Abbreviation/Symbol**	**Definition**
CE	Objective pair combining cost and emission	CL	Objective pair combining cost and loss
CV	Objective pair combining cost and voltage deviation	EL	Objective pair combining emission and loss
FACTS	Flexible AC transmission system devices	HV	Hypervolume performance indicator
IEEE	Institute of Electrical and Electronics Engineers	IGD	Inverted generational-distance indicator
LPSR	Linear reduction of population size	MEBO	Modified Effective Butterfly Optimizer
MO	Multi-objective formulation	MO-OPF/MOOPF	Multi-objective optimal power-flow formulation
MVAr	Reactive megavolt-ampere unit	MW	Megawatt unit
OPF	Optimal power-flow problem	PDF	Probability-density function
PV	Photovoltaic generation	p.u.	Per-unit representation
SHBA	Success history-based parameter adaptation	VAR	Volt-ampere reactive unit
VD	Voltage-deviation index	VPP	Virtual power-plant concept
WSMOPF	Weighted sum multi-objective OPF formulation	WT	Wind-turbine unit
*Ψ_s_*_,*c*_	Vector objective map for the multi-objective OPF model	*f_r_*(*x*,*u*), *M*	r-th scalar objective term and the number of objectives
*g*(·), *h*(·)	Vectors of equality and inequality constraints	*x*, *u*	State variables and controllable decision variables
*P_ref_*, *P_G_*_,*slack*_	Active generation assigned to the reference/slack bus	*V_PQ_*	Voltage magnitudes at load/PQ buses
*Q_G_*	Reactive-power outputs of generators	*S_br_*	Apparent-power loading of branches
*P_G_*, *V_G_*	Generator active-power dispatches and voltage set-points	*Q_sh_*, *τ*	Shunt VAR settings and transformer tap ratios
*ΔP_k_*, *ΔQ_k_*	Active and reactive power-balance residuals at bus k	*P_G_*_,*k*_, *Q_G_*_,*k*_	Generated active and reactive powers at bus k
*P_D_*_,*k*_, *Q_D_*_,*k*_	Active and reactive demand powers at bus k	*V_k_*, *V_r_*	Voltage magnitudes of buses k and r
*Y_kr_*, *θ_kr_*	Magnitude and angle of the admittance between buses k and r	*δ_k_*, *δ_r_*	Voltage phase angles for buses k and r
*N_b_*	Total bus count	*i*, *j*, *k*, *l*, *r*	Context-dependent indices for units, plants, buses, branches, or objectives
*G*, *T*, *C_sh_*	Generator, tap-changing transformer, and shunt VAR device sets	*L*, *B*, *D*	Load-bus, constrained-branch, and demand-bus sets; D also denotes MEBO dimension
*f_C_*, *f_E_*	Generation-cost and emission objectives	*f_PL_*, *f_VD_*	Real power loss and voltage-deviation objectives
*A_i_*, *B_i_*, *C_i_*	Quadratic cost-curve coefficients for generator i	*α_i_*, *β_i_*, *γ_i_*, *ζ_i_*, *ρ_i_*	Emission-model coefficients for generator i
*V_ref_*	Nominal reference voltage, set to 1.0 p.u.	*Y*	Constraint-output vector containing VPQ and QG
*w*, *n*	Penalty weight and number of constrained quantities	*UB_G_*_,*slack*_, *LB_G_*_,*slack*_	Upper and lower active-power limits of the slack generator
*UB_i_*, *LB_i_*	Upper and lower limits of the i-th constrained quantity	*λ*, *λ_i_*	Weighting coefficient in the weighted multi-objective functions
*f_i_^CL^*(*x*), *f_i_^CV^*(*x*)	Weighted cost–loss and cost–voltage objectives for weight index i	*f_i_^CE^*(*x*), *f_i_^EL^*(*x*)	Weighted cost-emission and emission–loss objectives for weight index i
*A*, *B* ∈ {*C*, *L*, *V*, *E*}	Objective labels used to select the two-objective case	*i_opt_*, *x_opt_*	Index and decision vector of the selected compromise solution
*dist_M_*	Distance value computed with metric M	*p*, *q*, *μ*, *S*	Vectors, mean vector, and covariance matrix used in distance evaluation
*PS_max_*, *PS_min_*, *PS*	Maximum, minimum, and current population sizes	*memSize*, *archSize*	Sizes of the parameter memory and archive
*FE_max_*	Maximum number of function evaluations	*F*, *CR*, *T*, *Freq*	MEBO step size, crossover probability, shape parameter, and frequency term
*probPerch*, *probPat*	Probabilities assigned to perching and patrolling phases	*c*, *k*	Weibull scale and shape parameters for wind speed
*v_in_*, *v_r_*, *v_out_*	Cut-in, rated, and cut-out wind speeds	*P_ws_*_,*j*_, *P_wav_*_,*j*_	Scheduled and available wind powers for wind plant j
*P_w_*_,*j*_, *p_w_*_,*j*_, *f_w_*(*p_w_*_,*j*_)	Wind-power variable, integration variable, and wind-power density function	*g_j_*	Direct cost coefficient for wind plant j
*K_Rw_*_,*j*_, *K_Pw_*_,*j*_	Reserve and penalty cost coefficients for wind plant j	*C_w_*_,*j*_, *C_Rw_*_,*j*_, *C_Pw_*_,*j*_	Direct, reserve, and penalty cost functions for wind plant j
*P_ss_*, *P_sav_*	Scheduled and available photovoltaic powers	*f_s_*	Solar-power probability-density function
*K_Rs_*, *K_Ps_*	Reserve and penalty coefficients of the photovoltaic plant	*C_Rs_*, *C_Ps_*	Reserve and penalty cost functions of the photovoltaic plant
*E*[·]	Expectation operator	*F_total_*	Total cost over thermal, wind, and photovoltaic components
*Cost_thermal_*, *Cost_wind_*, *Cost_PV_*	Thermal, wind, and photovoltaic cost components	*μ_s_*, *σ*	Mean and standard-deviation parameters of the lognormal solar model
*λ_ren_*_1_, *λ_ren_*_2_, *λ_ren_*_4_	Case-specific weights for renewable-integrated optimization cases	Δ	Spread metric
